# Self‐Degradable Nanogels Reshape Immunosuppressive Tumor Microenvironment via Drug Repurposing Strategy to Reactivate Cytotoxic CD8^+^ T Cells

**DOI:** 10.1002/advs.202301661

**Published:** 2023-05-05

**Authors:** Hao Tian, Wenxi Li, Guohao Wang, Ye Tian, Jie Yan, Songtao Zhou, Xinying Yu, Bei Li, Yunlu Dai

**Affiliations:** ^1^ Cancer Center and Institute of Translational Medicine Faculty of Health Sciences University of Macau Macau Macau SAR 999078 China; ^2^ MoE Frontiers Science Center for Precision Oncology University of Macau Macau Macau SAR 999078 China

**Keywords:** CD8^+^ T cells, drug repurposing, immunosuppressive tumor microenvironment, self‐degradable nanogels

## Abstract

Intratumoral CD8^+^ T cells are crucial for effective cancer immunotherapy, but an immunosuppressive tumor microenvironment (TME) contributes to dysfunction and insufficient infiltration. Drug repurposing has successfully led to new discoveries among existing clinical drugs for use as immune modulators to ameliorate immunosuppression in TME and reactivate T‐cell‐mediated antitumor immunity. However, due to suboptimal tumor bioavailability, the full potential of immunomodulatory effects of these old drugs has not been realized. The self‐degradable PMI nanogels carrying two repurposed immune modulators, imiquimod (Imi) and metformin (Met), are reported for TME‐responsive drug release. It remodels the TME through the following aspects: 1) promoting dendritic cells maturation, 2) repolarizing M2‐like tumor‐associated macrophages, and 3) downregulating PD‐L1 expression. Ultimately, PMI nanogels reshaped the immunosuppressive TME and efficiently promote CD8^+^ T cell infiltration and activation. These results support that PMI nanogels can potentially be an effective combination drug for enhancing the antitumor immune response of anti‐PD‐1 antibodies.

## Graphical abstract

Self‐degradable nanogels with tumor microenvironment (TME)‐responsiveness exhibit unique properties of targeted and rapid drug release, which can revert the immunosuppressive TME to the anti‐tumor state. And the improved immunosuppressive TME can reactivate T‐cell‐mediated antitumor immunity by promoting CD8^+^ T cell infiltration and activation, which enhances the anticancer activity of current cancer immunotherapy.

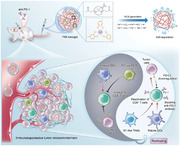

## 1 Introduction

CD8^+^ T cells have become a focus in tumor immunology research due to their potent tumor‐killing capability.^[^ ^1^ ^]^ It has been abundantly demonstrated that the success of cancer immunotherapy requires the infiltration and activation of CD8^+^ T cells.^[^ ^2^ ^]^ However, many patients do not benefit from current cancer immunotherapy because the immunosuppressive tumor microenvironment (TME) contributes to a lack of antitumor immune response of CD8^+^ T cells.^[^ ^3^ ^]^ Therefore, a therapeutic strategy to revert the immunosuppressive TME to reactivate cytotoxic CD8^+^ T cells may enhance the anticancer activity of current cancer immunotherapy.^[^ ^4^ ^]^

Drug repurposing involves using existing clinical drugs for new medical indications.^[^ ^5^ ^]^ Notably, old drugs that were approved for the long‐term treatment of common diseases such as hyperlipidemia, diabetes, and hypertension have been repurposed as immune modulators for alleviating immunosuppression in TME by activating innate sensing pathways or targeting immunosuppressive factors.^[^ ^6^ ^]^ Imiquimod (Imi) is commonly used as a topical drug against warts on the skin of the genital and anal areas but has recently been identified as a toll receptor 7 (TLR‐7) agonist that promotes dendritic cells (DCs) maturation.^[^ ^7^ ^]^ And the clinical trials in combination with anti‐PD‐1 antibodies in the treatment of metastatic melanoma (NCT03276832) revealed enormous application potential.^[^ ^8^ ^]^

Similarly, metformin (Met) is used in the treatment of type 2 diabetes as a first‐line drug, and exerts immunomodulatory effects by activating adenosine 5′‐monophosphate (AMP)‐activated protein kinase (AMPK).^[^ ^9^ ^]^ M2‐like tumor‐associated macrophages (TAMs) are immunosuppressive cells that suppress T‐cell function directly or indirectly, ultimately promoting tumor growth. Met can repolarize the tumor‐promoting M2 phenotype to the tumoricidal M1 phenotype by regulating the AMPK‐NF‐*κ*B signaling pathway. A high M1/M2 TAM ratio indicates an improved immunosuppressive TME and is associated with the enhanced antitumor immune response of CD8^+^ T cells.^[^ ^10^ ^]^ Meanwhile, Cha et al. proposed that Met‐activated AMPK can downregulate the expression of programmed death ligand‐1 (PD‐L1) in cancer cells by inducing abnormal degradation, which subsequently increases CD8^+^ T cell activity.^[^ ^11^ ^]^ Moreover, several clinical trials are ongoing to investigate the antitumor effect of Met combinations in cancer immunotherapy.^[^ ^12^ ^]^

**Figure 1:**
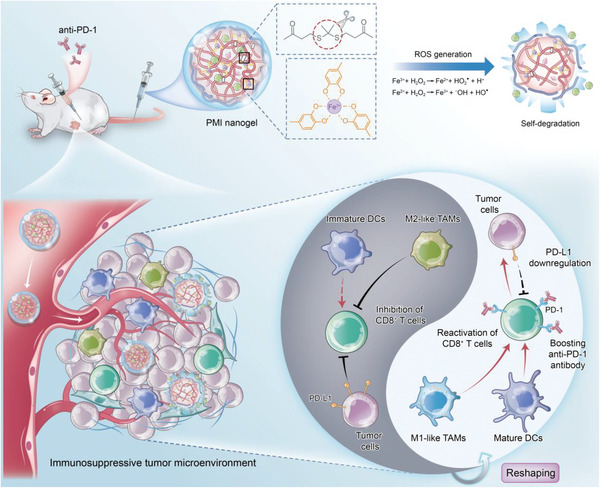
Illustration of self‐degradable PMI nanogels reshaping immunosuppressive TME to reactivate cytotoxic CD8^+^ T cells for anti‐PD‐1 antibody. After intravenous injection, PMI nanogels gradually accumulate in the tumor site and exhibit self‐degradation under the generation of ROS. TME‐responsive drug release alleviates immunosuppression in the TME by 1) promoting DCs maturation, 2) repolarizing M2‐like TAMs, and 3) downregulating PD‐L1 expression. As a result, the improved immunosuppressive TME promotes the infiltration and the activation of CD8^+^ T cells, which means PMI nanogels are potentially effective as a combination drug for current cancer immunotherapy.

However, there are inherent defects in the systemic administration of small molecules, including suboptimal tumor bioavailability, poor pharmacokinetics, and off‐target side effects. Thus, the full potential of the immunomodulatory effects of these old drugs has not been realized. Herein, we developed the self‐degradable nanogels (PMI nanogels) with TME‐responsive, which possesses unique properties of reactive oxygen species (ROS)‐specific activation and subsequent rapid drug release (**Figure** **1**). The nanogels consist of a ROS‐responsive amphipathic phenolic polymer. The hydrophobic terminal of the polymer was cross‐linked with the thioketal linker and conjugated with Met as a prodrug by forming acid‐sensitive imine bonds. Then, the PMI nanogels were formed via metal‐phenolic coordination, cross‐linking of polymer chains, and hydrophobic interaction when mixed with Fe^3+^ and Imi. After intravenous injection, PMI nanogels exhibited self‐degradation in TME due to the ROS generated by iron‐mediated catalytic reactions, and subsequently reverted the immunosuppressive TME to the anti‐tumor state by following aspects: 1) promoting DCs maturation, 2) repolarizing M2‐like TAMs, and 3) downregulating PD‐L1 expression. Ultimately, PMI nanogels improved immunosuppressive TME, which can reactivate CD8^+^ T cells’ antitumor immune response by promoting infiltration and the activation of CD8^+^ T cells. Moreover, the reactivation of cytotoxic CD8^+^ T cells supports PMI nanogels as a potentially effective combination drug for current cancer immunotherapy.

## 2 Results and Discussion

### 2.1 Synthesis and Characterization of PMI Nanogels

**Figure 2:**
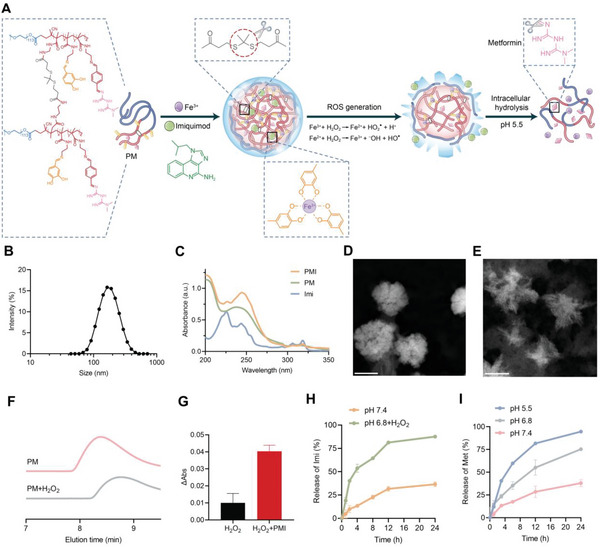
Characterization of PMI nanogels. A) Illustration of the preparation and degradation processes of PMI nanogels. B) Dynamic light scattering (DLS) result of PMI nanogels. C) The UV–vis absorption spectra of different components in PMI nanogels. D,E) Representative transmission electron microscopy (TEM) images of PMI in D) PBS and E) pH 6.8 solution with 100 µM H_2_O_2_ (scale bar: 200 nm). F) Polymer degradation triggered with 100 µM H_2_O_2_ measured by gel permeation chromatography (GPC). G) The absorbance change of DPBF to confirm the ROS generation. H) Imi release profiles within 24 h in PBS and pH 6.8 solution with 100 µM H_2_O_2_. I) Met release profiles within 24 h in different pH solutions (5.5, 6.8, and 7.4). Data are presented as mean values ± SD (*n* = 3).

ROS‐responsive phenolic polymer PM was first synthesized (**Figure** **2**A). The synthetic route for the block copolymer (PEG‐b‐NH_2_) was realized via RAFT polymerization according to our reported method.^[^ ^13^ ^]^ We subsequently introduced the thioketal linker on PEG‐NH_2_ through highly reactive acyl chlorides and free amino groups for rapid cleavage by ROS. The cross‐linked polymer was then conjugated with Met and 3,4‐dihydroxybenzaldehyde on its hydrophobic side chains via acid‐sensitive imine bonds.

The chemical structures of all intermediates and the final product were confirmed by proton nuclear magnetic resonance (^1^H NMR) spectroscopy (Figures S1–S7, Supporting Information). The mass ratio of Met in PM was calculated as 18.7% according to the peak (7.37–7.55 ppm) of Met in the ^1^H NMR. After mixing Fe^3+^ and Imi with PM under sonication, the final PMI nanogels were formed with an average hydrodynamic diameter of ≈200 nm (Figure 2B). The UV–vis absorption results also verified the successful fabrication of PMI, and the mass ratio of Imi in PMI was calculated to be 3.9% based on its characteristic absorption peak at 318 nm (Figure 2C and Figure S8, Supporting Information). Transmission electron microscopy (TEM) and energy dispersive X‐ray spectroscopy (EDS) mapping images of PMI showed spherical morphology and the successful coordination between dihydroxyphenyls in PM and Fe^3+^ ions (Figure 2D and Figure S10, Supporting Information).

### 2.2 TME‐Responsive Degradation and Drug Release Behaviors

To investigate the self‐degradation of PMI nanogels in TME, we prepared a pH 6.8 solution containing 100 µM H_2_O_2_ to mimic TME and incubated it with PMI nanogels for 6 h.^[^ ^14^ ^]^ Then, a TEM image was used to verify that the previously well‐assembled structures of PMI were broken (Figure 2E). This can be ascribed to the ROS‐responsive behavior of PM, which was confirmed by the right shift of H_2_O_2_‐treated retention time measured by gel permeation chromatography (GPC) (Figure 2F). In addition, the ROS generated by iron‐mediated catalytic reactions can accelerate the degradation process. The ROS generation was confirmed using 1,3‐diphenylisobenzofuran (DPBF), a highly specific probe. Upon the co‐incubation of PMI and H_2_O_2_, ROS generation was four times higher in comparison with H_2_O_2_ alone group (Figure 2G). The self‐degradation of PMI ultimately resulted in rapid drug release. More than 50% release of Imi was observed within 4 h after incubation with pH 6.8 solutions containing 100 µM H_2_O_2_ (Figure 2H and Figure S11, Supporting Information)_._ Next, we evaluated the hydrolysis rate of Met in different pH conditions by UV–vis absorption because of the acid‐labile imine groups (Figure 2I and Figure S12, Supporting Information). The acidity‐specific activation contributes to more than 80% Met release from PMI at pH 5.5 in comparison with about 50% release at pH 6.8 and negligible release at pH 7.4.

### 2.3 PMI Nanogels Efficiently Downregulated PD‐L1 Expression

**Figure 3:**
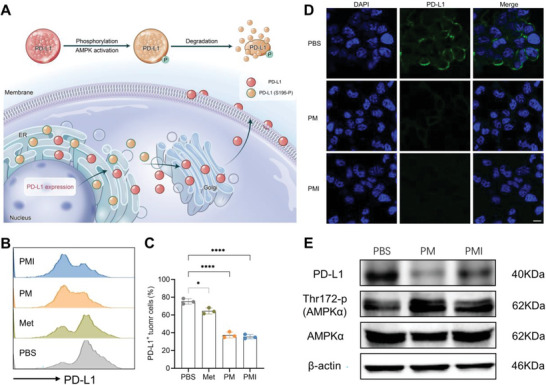
PMI nanogels downregulated PD‐L1 expression in tumor cells. A) Met‐activated AMPK promotes PD‐L1 degradation. B,C) Flow cytometric analysis and quantitative results of PD‐L1 expression. D) Confocal microscopy images of the PD‐L1 expression. Staining the nucleus with DAPI (blue). Scale bar: 10 µm. E) Western blot analysis of the association between AMPK activation and PD‐L1 downregulation. Data are presented as mean values ± SD (*n* = 3). Statistically significant differences between groups were identified by one‐way ANOVA. *p < 0.05; **p < 0.01; ***p < 0.001; ****p < 0.0001 are considered as statistically significant.

PD‐L1 is a well‐known immune checkpoint molecule that is exploited by cancer cells to shut down the antitumor immune responses of CD8^+^ T cells. According to a previous report, Met‐activated AMPK can promote PD‐L1 degradation by abnormally phosphorylating the PD‐L1 on S195 (**Figure** **3**A).^[^ ^11^ ^]^ 4T1 cells were stimulated with interferon‐*γ* (IFN‐*γ*) for 12 h and then incubated with PBS, free Met, PM, and PMI nanogels for 24 h, followed by a flow cytometry analysis of PD‐L1 expression (Figure 3B,C). Since the met concentration (20 µg mL^−1^) that we used was lower than previously reported, only weak inhibition of PD‐L1 was observed in the free Met group. However, the PM and PMI showed an obvious inhibition of PD‐L1 expression, which is possibly due to significantly increased cellular uptake of the PEGylated Met prodrug. Thus, to verify the cell uptake state by flow cytometry, we synthesized a new polymer, PM‐IR, which contained fluorescent dye IR 780 (Figure S13, Supporting Information). This polymer exhibited prominent uptake within 6 h (Figure S14, Supporting Information).

PMI‐induced PD‐L1 downregulation was confirmed by confocal laser scanning microscopy (CLSM) using PE‐conjugated PD‐L1 monoclonal antibody (Figure 3D). We also used western blot assay to investigate the detailed molecular mechanism of the association between AMPK*α* and PD‐L1 undergoing Met treatment. As shown in Figure 3E, the PM or PMI treatments significantly augmented the phosphorylation of AMPK*α* (Thr172) concomitantly with the downregulation of PD‐L1 (Figure 3E). Notably, PMI efficiently downregulated PD‐L1 expression at a low concentration of Met. When the experiments were performed at a relatively high concentration level, PMI directly caused cell apoptosis (Figure S15, Supporting Information).

### 2.4 PMI Nanogels Induced DCs Maturation and Macrophages Repolarization

**Figure 4:**
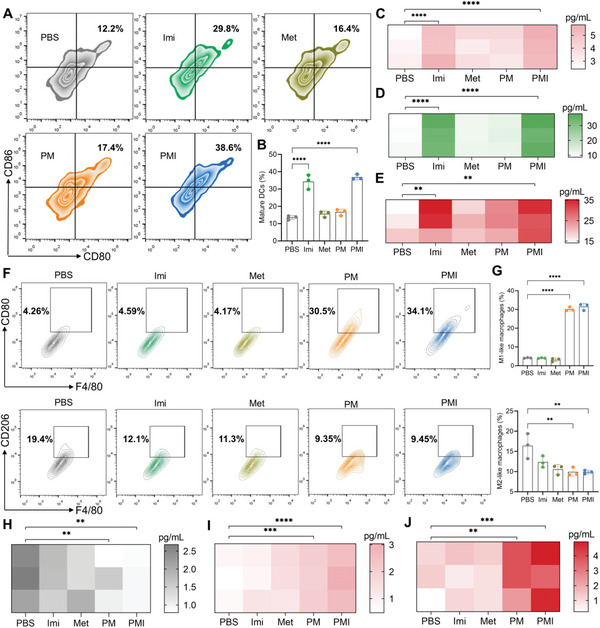
PMI nanogels promote DCs maturation and macrophage repolarization in vitro. A,B) Flow cytometry and quantitative analysis of mature DCs. C–E) The heat maps of the concentration of C) IL‐6, D) IL‐12, and E) TNF‐*α* in the supernatants after various treatments. F,G) Flow cytometry and quantitative analysis of macrophage repolarization. H–J) The heat maps of the concentration of H) IL‐10, I) IL‐6, and J) TNF‐*α* in the supernatants after various treatments. Data are presented as mean values ± SD (*n* = 3). Statistically significant differences between groups were identified by one‐way ANOVA. *p < 0.05; **p < 0.01; ***p < 0.001; ****p < 0.0001 are considered as statistically significant.

Mature DCs are crucial innate immune cells responsible for T cell activation, but most of the DCs in the TME are immature, which inhibits the T cell‐mediated antitumor responses. Therefore, we isolated bone marrow cells from BALB/c mice to investigate whether Imi (a TLR7 agonist) encapsulated in PMI nanogels has the capability of promoting DCs maturation. Briefly, the bone marrow‐derived DCs (BMDCs) obtained were treated with PBS, Imi, Met, PM, or PMI for 48 h and collected for flow cytometry analysis (CD11c^+^CD80^+^CD86^+^ gated on CD11c^+^ cells). The results are shown in **Figure** **4**A,B. In the groups with Imi, the maturation of BMDCs was obviously triggered. Particularly, the PMI group reached the highest maturation level, which was markedly increased to 36.8 ± 1.5%, whereas only 13.5 ± 1.2% of mature BMDCs were found in the PBS group. Consistent with the DCs maturation trend, pro‐inflammatory cytokines secreted by BMDCs, including IL‐6, IL‐12, and TNF‐*α*, also exhibited significant increases after various treatments (Figure 4C–E).

Similar to the immature DCs, M2‐like macrophages are immunosuppressive cells that suppress T‐cell function directly or indirectly. We verified the effects of Met in PMI on macrophage repolarization. Firstly, RAW264.7 cells were treated with 20 ng mL^−1^ of IL‐4 to obtain M2‐like macrophages. The cells were given different treatments to analyze the PMI's impact on macrophage repolarization through flow cytometry (M1: F4/80^+^CD86^+^, M2: F4/80^+^CD206^+^) (Figure 4F,G). Compared with the PBS group, PM and PMI treatments significantly increased the proportion of M1‐like macrophages to 30.2 ± 1.1% and 31.5 ± 1.8%, respectively, while decreasing the proportion of M2‐like macrophages. The reason is the Met‐mediated activation of the AMPK signaling pathway. The repolarization of macrophages mediated by Met was also investigated by the corresponding secretion of cytokines, including IL‐10, IL‐6, and TNF‐*α* (Figure 4H–J). All results suggested that PMI may ameliorate the immunosuppressive TME by promoting DCs maturation and repolarizing M2‐like macrophages.

### 2.5 PMI Nanogels Reactivated Cytotoxic CD8^+^ T Cells in Co‐Implanted Tumors

**Figure 5:**
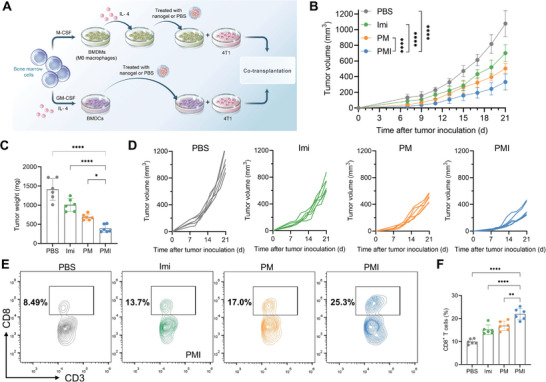
PMI nanogels reactivated cytotoxic CD8^+^ T cells in a co‐implanted model. A) illustration of the immune cells and tumor cells co‐transplantation. B) Average tumor growth curves. C) The tumor weight analysis. D) The individual tumor growth curves. E,F) Flow cytometric and quantitative analyses of CD8^+^ T cells. Data are presented as mean values ± SD (*n* = 6). Statistically significant differences between groups were identified by one‐way ANOVA in (C,F) or two‐way ANOVA in (B). *p < 0.05; **p < 0.01; ***p < 0.001; ****p < 0.0001 are considered as statistically significant.

Cytotoxic CD8^+^ T cells are the most powerful effectors in the anticancer immune responses, but in the immunosuppressive TME, The T‐cell‐mediated antitumor immunity is restricted by the action of immunosuppressive cells, such as immature DCs, M2‐like macrophages and regulatory T cells (Tregs). Encouraged by the successful modulation of immature DCs and M2‐like macrophages in vitro, we assumed that these pro‐inflammatory immune cells could ameliorate the immunosuppressive TME to reactivate cytotoxic CD8^+^ T cells. Bone marrow cells from mice were isolated and differentiated into murine BMDCs. When not activated, they are immature and can resemble intratumoral DCs. Meanwhile, bone marrow‐derived macrophages (BMDMs) were also stimulated with IL‐4 to obtain the M2 phenotype. The obtained BMDCs and BMDMs were pretreated with different formulations and co‐implanted into breast pads with 4T1 tumor cells (**Figure** **5**A).

Based on the treatment process, immature DCs, M2‐like macrophages, and tumor cells made up the PBS group. As the results in Figure 5B–D show, the tumor growth rate in the PBS group was incredibly fast, which may be related to the support of the tumor growth by the immunosuppressive cells. In other treatment groups, Imi can cause BMDCs to mature. However, the presence of M2‐like BMDMs continued to promote tumor growth and directly or indirectly reduce the effectiveness of anti‐tumor cytotoxicity, resulting in a slight inhibition in tumor growth.

There were problems in the treatment of the PM group. The M1‐like BMDMs alone failed to achieve the same therapeutic results as PMI. In contrast, all the immune cells were activated after PMI treatment and tumor growth was significantly delayed. Subsequent flow cytometry analysis of the infiltration and the activation of CD8^+^ T cells confirmed our hypothesis. The PMI‐pretreated group exhibited a rate of intratumor infiltrating CD8^+^ T cells (CD45^+^CD3^+^CD8^+^) of ≈21.9 ± 2.5%, which was around 2.20 times higher than that of the PBS group (Figure 5E,F).

The activated state of CD8^+^ T cells was analyzed by flow cytometry. According to the results, the PBS group had the lowest level of cytotoxic marker expression (IFN‐*γ* ^+^ and GzmB^+^). However, the expression of markers was increased in the Imi and PM groups. This demonstrated that the mature DCs and M1‐like TAMs were beneficial for reshaping the immunosuppressive TME, which can prevent CD8^+^ T cells from becoming exhausted and having impaired functions. It's not surprising that the combined PMI group exhibited the highest expression of cytotoxic markers (Figure S16, Supporting Information).

### 2.6 PMI Nanogels Sufficiently Reshaped the Immunosuppressive TME to Reactivate Cytotoxic CD8^+^ T Cells

Before exploring the biological function of PMI in vivo, we evaluated its biodistribution in a subcutaneous 4T1 tumor model by fluorescence imaging. The results showed that PMI gradually accumulated in tumors after 2 h post‐injection and reached a maximum level at 12 h. Intense fluorescence within 48 h indicated long intertumoral retention, suggesting potential for remodeling immunosuppressive TME (Figure S17, Supporting Information).

**Figure 6:**
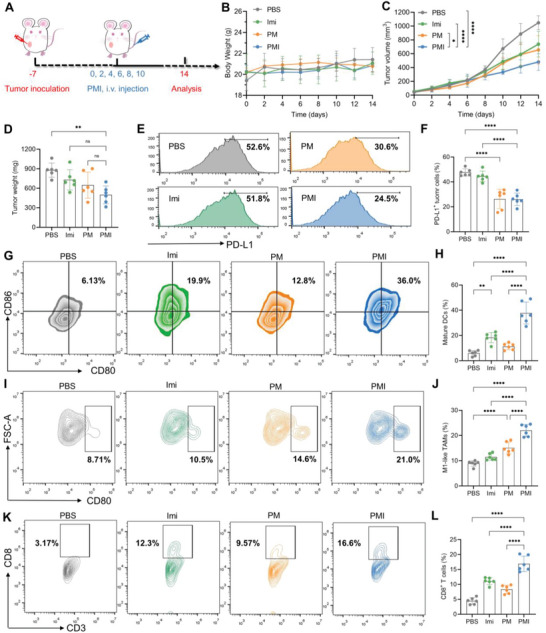
PMI nanogels sufficiently reshaped the immunosuppressive TME to reactivate cytotoxic CD8^+^ T cells. A) Tumor therapeutic scheme in vivo. B) Body weight variations of mice. C) Average tumor growth curves. D) Tumor weight analysis. E,F) Flow cytometry and quantitative analyses of tumoral PD‐L1 expression after different treatments. G,H) Flow cytometry and quantitative analyses of mature DCs in TDLNs. I,J) Flow cytometry and quantitative analyses of M1‐like TAMs in TME after different treatments. K,L) Flow cytometry and quantitative analyses of CD8^+^ T cells in TME. Data are presented as mean values ± SD (*n* = 6). Statistically significant differences between groups were identified by one‐way ANOVA in (D–L) or two‐way ANOVA in (C). *p < 0.05; **p < 0.01; ***p < 0.001; ****p < 0.0001 are considered as statistically significant.

We then assessed the reshaped immunosuppressive TME induced by PMI nanogels in an orthotopic breast cancer model. We inoculated 4T1 cells into the left breast pad of female BALB/c mice. At 7 days post‐inoculation (termed as day 0), the mice were randomly divided and subjected to various treatments (*n* = 6). As illustrated in **Figure** **6**A, Imi (3.75 mg kg^−1^) and Met (15 mg kg^−1^) were injected intravenously at equal concentrations with different formulations once every 2 days for a total of six times. During the treatment period, we monitored the mice's weight and tumor volume every 2 days. There were negligible body weight variations over the experimental duration (Figure 6B). We also carried out a biosafety evaluation by evaluating the chemistry parameters in serum (Figure S18, Supporting Information). The results indicate the biosafety of PMI. On day 14, we sacrificed the mice and collected tumors. Tumor growth curves and tumor weights show that PMI nanogels had the best anti‐tumor efficacy. Imi or Met alone showed similar tumor inhibition effects, and only slightly delayed tumor growth compared with the untreated group (Figure 6C,D and Figure S19, Supporting Information). The lack of obvious significance between treated groups in tumor weight may be attributed to the fact that both drugs are used in a drug‐repurposing strategy, that focuses on the regulation of the immunosuppressive TME rather than directly inhibiting tumors.

According to the in vitro results, we first determined the situation of PD‐L1 expression in cancer cells. The results showed that PMI nanogels reduced PD‐L1 expression from ≈50% to 20% compared to the PBS group (Figure 6E,F). To investigate the DCs maturation (CD11c^+^CD80^+^CD86^+^), tumor‐draining lymph nodes (TDLNs) were also collected. As shown in Figure 6G,H, the maturation rate of the PBS group was 5.89 ± 2.18%, and PMI treatment dramatically increased the frequencies of mature DCs (37.8 ± 8.7%) compared with the results of the PBS group.

The ELISA assay was also carried out to investigate the secretion level of cytokines, which indicated that the pro‐inflammatory cytokines IL‐6, IL‐12, and TNF‐*α* were increased after the treatment. In contrast, the anti‐inflammatory cytokine IL‐10 was decreased by 65.9% compared to the PBS group (Figure S20, Supporting Information). The decrease in the level of anti‐inflammatory factors could also indicate a situation of repolarization of tumor‐promoting M2‐like TAMs (CD11b^+^F4/80^+^CD206^+^) to the tumoricidal M1 phenotype (CD11b^+^F4/80^+^CD80^+^) in TME. Consistent with the results in vitro, PMI significantly increased the M1/M2 TAM ratio (Figure 6I,J and Figure S22, Supporting Information). All these results implied the effective regulation of the immunosuppressive TME by PMI nanogel, which might create a more favorable environment for T cells. As expected, the PBS group only showed negligible tumor‐infiltrating CD8^+^ T cells (4.44 ± 1.10%) while the PMI group showed a significant increase of CD8^+^ T cell infiltration to 16.8 ± 2.6%. This suggests the efficient enhancement of antitumor immunity after ameliorating the tumor immune microenvironment (Figure 6K,L).

M2‐like TAMs are closely related to regulatory T cells (Tregs, CD45^+^CD3^+^CD4^+^Foxp3^+^) infiltration, so PMI treatment‐induced M1‐like TAMs repolarization could lead to a notable decrease of Tregs (Figure S23, Supporting Information). The CD8^+^/ Treg ratio in the PMI group was also remarkably increased compared with other groups, indicating improved cytotoxicity of tumor‐infiltrating CD8^+^ T cells (Figure S24, Supporting Information).

### 2.7 PMI Nanogels Enhanced Therapeutic Efficiency of Anti‐PD‐1 Antibody by Reactivating Cytotoxic CD8^+^ T Cells

**Figure 7:**
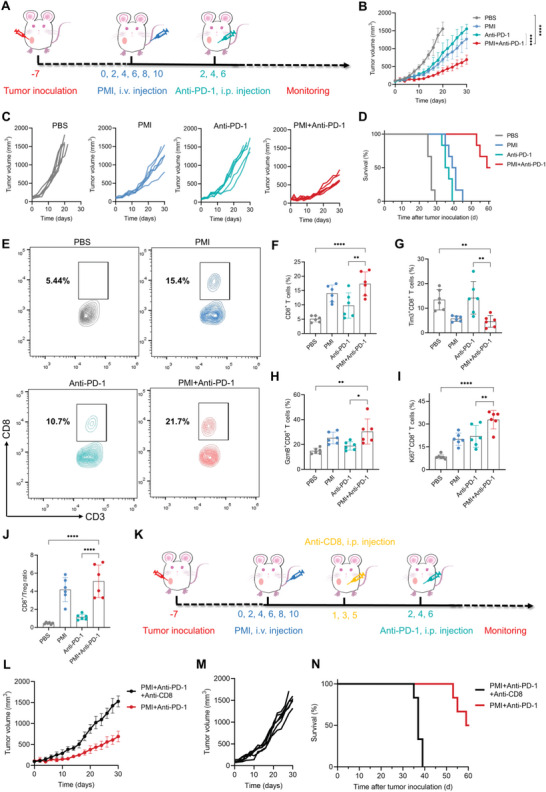
PMI nanogels enhanced therapeutic efficiency of anti‐PD‐1 antibodies by reactivating cytotoxic CD8^+^ T cells. A) Illustration of the combination experiment of PMI nanogels and anti‐PD‐1 antibody. B) Average tumor growth curves. C) Individual tumor growth curves. D) Survival curves of tumor‐bearing mice under different treatments. E,F) Flow cytometry and quantitative analyses of CD8^+^ T cells. G–I) Flow cytometry analysis of G) Tim 3^+^, H) GzmB^+^, and I) Ki67^+^ in CD8^+^ T cells. J) Quantitation of the intratumoral ratio of CD8^+^ T cells to Treg. K) Illustration of the antitumor experiment while combining in vivo CD8^+^ T cell depletion. L) Tumor growth curves with CD8^+^ T cell depletion. M) Individual tumor growth curves with CD8^+^ T cell depletion. N) Survival rates of mice with CD8^+^ T cell depletion. Data are presented as mean values ± SD (*n* = 6). Statistically significant differences between groups were identified by one‐way ANOVA in (F–I) or two‐way ANOVA in (B). *p < 0.05; **p < 0.01; ***p < 0.001; ****p < 0.0001 are considered as statistically significant.

In consideration of reactivating cytotoxic CD8^+^ T cells in the reshaped TME, we hypothesized that combination with PMI nanogels would enhance the therapeutic efficiency of anti‐PD‐1 antibody. Therefore, the corresponding combination's therapeutic effects were verified using the treatment procedure shown in **Figure** **7**A. The dose of administered anti‐PD‐1 antibody was 100 µg per mouse every 2 days for a total of three times. There was no obvious body weight loss in either group during the entire treatment period (Figure S25, Supporting Information), and the serum parameters showed no significant difference in the various groups, revealing the biosafety of PMI nanogels for combination treatment (Figure S26, Supporting Information).

The tumor volume of each mouse was monitored for 30 days (Figure 7B,C). Compared with the PBS group, those treated with PMI or anti‐PD‐1 antibody alone only showed a slight inhibitory effect on tumor growth. In contrast, the combination treatment exhibited a superior antitumor efficiency which inhibited tumor proliferation and delayed tumor growth. In addition, the persisting tumor growth inhibition promoted the overall survival time. As shown in Figure 7D, all mice that received anti‐PD‐1 antibody or PMI treatment successively died within 45 days, showing negligible survival benefits compared to the PBS group. In contrast, the combination group showed prolonged survival of tumor‐bearing mice, with 50% of mice surviving over 60 days.

We then repeated the same therapeutic experiment and sacrificed the mice on the 14^th^ day to collect serum, tumors, and TDLNs for immune analysis. The ELISA results showed that the level of pro‐inflammatory cytokines was increased in the combined treatment group (Figure S27, Supporting Information). Subsequently, DCs maturation was verified by flow cytometry (Figure S28, Supporting Information). The anti‐PD‐1 antibody alone was also insufficient for DC maturation (21.0 ± 3.0%). Compared to PBS (13.2 ± 3.6%), the anti‐PD‐1 antibody alone was also insufficient for DC maturation (21.0 ± 3.0%), but there was a high rate of mature DCs in the combination group (32.4 ± 4.9%). Anti‐PD‐1 antibodies also exhibited negligible modulation of repolarization of M2 to M1‐like TAMs, and the M2‐like TAMs were still the dominant phenotype in tumors (40.0 ± 9.3%). These results implied that the immunosuppressive TME leads to resistance to anti‐PD‐1 antibodies. In contrast, the combination group also showed a significant increase in M1‐like TAMs and a marked decrease in M2 phenotype when compared with the anti‐PD‐1 antibody group (Figure S29, Supporting Information).

In view of the reshaped immunosuppressive TME reactivating CD8^+^ T cells’ antitumor immune response, we assumed that the promoted infiltration and the activation of CD8^+^ T cells were the reason for the enhanced therapeutic efficiency of anti‐PD‐1 antibody. Therefore, the rate of intratumor infiltrating CD8^+^ T cells was determined by flow cytometry (Figure 7E,F). Obviously, the combination treatment resulted in the most significant CD8^+^ T cells’ infiltration (17.4 ± 4.2%), which was 3.99‐ and 2.03‐fold higher than that of PBS and anti‐PD‐1 groups, respectively. We then evaluated T‐cells’ states after the treatment since long‐term exposure to immunosuppressive TME can convert CD8^+^ T cells into exhausted cells and impair their functions. TIM3 is a key marker of T cell exhaustion, and the PBS and anti‐PD‐1 groups showed a high proportion of Tim3^+^CD8^+^ T cells among all infiltrated CD8^+^ T cells. That of the combination group was significantly reduced by half compared to the PBS group (Figure 7G and Figure S30, Supporting Information). Moreover, GzmB and Ki67 are markers of activation and proliferation and also exhibited the highest rate of GzmB^+^CD8^+^ and Ki67^+^CD8^+^ T cells in the combination group: 30.4 ± 10.1% and 32.9 ± 6.1%, respectively (Figure 7H,I). The decrease in Treg population (Figure S31, Supporting Information) resulted in the CD8^+^T/Treg ratio being remarkably increased in the combination group (Figure 7J). All results strongly supported the hypothesis that PMI nanogels enhanced the therapeutic efficiency of anti‐PD‐1 antibodies by reactivating cytotoxic CD8^+^ T cells in the reshaped TME.

To demonstrate the involvement of CD8^+^ T cells in boosting the efficacy of anti‐PD‐1 antibody, the CD8^+^ T cell‐depleted mice subjected to the same combination treatment were regarded as a control group. They administered the anti‐CD8 antibodies 1 day before the anti‐PD‐1 antibodies and administered three doses every 2 days (Figure 7K). As expected, the depletion of CD8^+^ cells impaired the antitumor effect, which did not exhibit significant tumor inhibition (Figure 7L,M). The tumors kept growing steadily during the treatment, resulting in overall survival of fewer than 40 days (Figure 7N). Collectively, all results indicated that PMI nanogels’ enhancement of the therapeutic effects of anti‐PD‐1 antibody was dependent on the presence of CD8^+^ T cells.

## 3 Conclusion

Drug repurposing is a viable strategy that has the potential to successfully bring new hope to cancer treatment. Although these existing drugs were not originally designed for cancer treatment, reasonable medication use can be realized according to the relevant signaling pathways. However, the systemic administration of these drugs may suffer from suboptimal tumor bioavailability, poor pharmacokinetics, and off‐target side effects. In order to realize the full potential of these older drugs, the existence of an effective therapeutic solution is inevitable. Here, we have provided a strategy for supporting drug repurposing via nanotechnology. In summary, we developed self‐degradable nanogels with TME‐responsive release performance for loading two repurposed drugs (Imi and Met) as immune modulators to reshape the immunosuppressive TME and benefit current cancer immunotherapy. Both in vitro and in vivo results validated that the PMI nanogels efficiently remodeled the immunosuppressive TME by following aspects: 1) promoting DCs maturation, 2) repolarizing M2‐like TAMs to M1‐like TAMs, and 3) and downregulating PD‐L1 expression. Ultimately, we found that the reshaped TME efficiently reactivated T‐cell‐mediated antitumor immunity by enhancing the activation and infiltration of CD8^+^ T cells. The results of reactivation of cytotoxic CD8+ T cells support PMI nanogels as a potentially effective combination drug for current cancer immunotherapy.

## 4 Experimental Section

### Preparation of the PMI Nanogels

A solution of deionized water and methanol (5/1, v/v) was first prepared. 6 mg of PM, 1 mg of FeCl_3_, and 1 mg of Imi were all dissolved in methanol (1 mL). Subsequently, all materials were added to the solution under sonication. For use and storage, the methanol was removed by vacuum suction, the rest of the solution was purified by centrifugation at 5000 g for 20 min to separate unencapsulated components and it was filtrated with a 220 nm filtration membrane. Finally, the concentrated solution was stored at 4 °C.

### Self‐Degradable Properties in Response to the TME

The condition of TME was first simulated using a pH 6.8 solution with 100 µM H_2_O_2_. Then PMI nanogels solution was diluted in it and stirred at room temperature for 6 h. The differences in the morphology of PMI in PBS and pH 6.8 solution with 100 µM H_2_O_2_ solution were determined by TEM images. In another experiment, under the same conditions, the samples were taken from the solution at 0, 1, 3, 6, 12, and 24 h to measure the drug release by UV–vis absorption. The characteristic peaks of Imi and Met for calculation were 318 nm and 245 nm, respectively.

### Downregulation of PD‐L1 In Vitro

IFN‐*γ* (50 ng mL^−1^) was used to stimulate 4T1 cells overnight to induce PD‐L1 expression in all experiments. Then, all of the cell media was replaced by different formulations at the same Met concentrations (10 µg mL^−1^) and cultured for 1 day.

### DCs Maturation In Vitro

A BALB/c mouse (8‐10 weeks old) was used to isolate bone marrow cells and cultured with IL‐4 (10 ng mL^−1^) and GM‐CSF (20 ng mL^−1^) to obtain BMDCs. Then these immature DCs were treated with PBS, free Imi and Met, PM, and PMI at the Imi (4 µg mL^−1^) and Met (20 µg mL^−1^). After treatment for 1 day, the cells were harvested and analyzed by flow cytometry.

### Repolarization of M2 Macrophages In Vitro

RAW 264.7 was considered as M0 macrophages. Then, the cells were stimulated with IL‐4 (20 ng mL^−1^) for 1 day to obtain M2‐like macrophages. After that, the cells were treated with PBS, free Imi and Met, PM, and PMI at the Imi (4 µg mL^−1^) and Met (20 µg mL^−1^). Then the cells were harvested after 24 h and the expression of M1 or M2‐related marker were evaluated by flow cytometry.

### Immune Cells and Tumor Cells Co‐Transplantation

Immature BMDCs were obtained as mentioned. Bone marrow cells were cultured with 20 ng mL^−1^ M‐CSF for 5 days to obtain macrophages (BMDMs). The resulting BMDMs were M0 macrophages, and M2‐like macrophages were obtained by stimulation for 1 day with 20 ng mL^−1^ IL‐4. Then, the immature BMDCs and M2‐like BMDMs were treated with PBS or PMI (equal to 100 µg mL^−1^ PM) for 24 h, and orthotopically co‐implanted with 4T1 cells at a ratio of 1:1:1 (1 × 10^6^ tumor cells per mouse).

### Biodistribution

All animal procedures were performed using an approved protocol (UMARE‐030‐2018) by the University of Macau Animal Ethics Committee. To investigate the in vivo distribution, PMI was prepared by using IR780‐labeled PM. Then, tumor‐bearing mice were intravenously injected with 150 µL of PMI (equal to 1 mg kg^−1^ IR780). Fluorescent (FL) imaging was obtained by an in vivo imaging system (IVIS, Ex/Em = 640/720 nm) at different time points. At 12 h post‐injection, the major organs and tumors in another repeated experiment were collected for ex vivo imaging.

### Tumor Treatment Process

Mice were intravenously administrated PBS, Imi (delivered by the same polymer but without Met), PM, and PMI equivalent to an Imi dosage of 3.75 mg kg^−1^ or Met dosage of 15 mg kg^−1^ every 2 days (six times in total). Anti‐PD‐1 antibody (100 µg per mouse) was administered by intraperitoneal injections every 2 days three times. For CD8 depletion studies, anti‐CD8 (10 mg kg^−1^) was administered 1 day prior to anti‐PD‐1 treatment and then every 2 days for a total of three doses. When the tumor volume exceeded 1500 mm^3^, the mice were sacrificed.

### Statistical Analysis

All quantitative data were presented as mean ± SD. Software GraphPad Prism 9.0.0 was used for the data analysis. For multiple comparisons, one‐way ANOVA or two‐way ANOVA was used. Statistical analysis was performed as *p < 0.05, **p < 0.01, ***p < 0.001 and ****p < 0.0001.

## Conflict of Interest

The authors declare no conflict of interest.

## Supporting information

Supporting Information

## Data Availability

The data that support the findings of this study are available from the corresponding author upon reasonable request.

## References

[1] V. Appay , D. C. Douek , D. A. Price , Nat. Med. 2008, 14, 623. 18535580 10.1038/nm.f.1774

[2] a) S. L. Meier , A. T. Satpathy , D. K. Wells , Nat. Cancer 2022, 3, 143; 35228747 10.1038/s43018-022-00335-8

[3] a) T. L. Whiteside , Oncogene 2008, 27, 5904; 18836471 10.1038/onc.2008.271 PMC3689267

[4] a) S. L. Shiao , A. P. Ganesan , H. S. Rugo , L. M. Coussens , Genes Dev. 2011, 25, 2559; 22190457 10.1101/gad.169029.111 PMC3248678

[5] a) C. R. Chong , D. J. Sullivan , Nature 2007, 448, 645; 17687303 10.1038/448645a

[6] a) L. Sleire , H. E. Førde , I. A. Netland , L. Leiss , B. S. Skeie , P. Ø. Enger , Pharmacol. Res. 2017, 124, 74; 28712971 10.1016/j.phrs.2017.07.013

[7] a) M. A. Stanley , Clin. Exp. Dermatol. 2002, 27, 571; 12464152 10.1046/j.1365-2230.2002.01151.x

[8] S. Y. van der Zanden , J. J. Luimstra , J. Neefjes , J. Borst , H. Ovaa , Trends Immunol. 2020, 41, 493. 32381382 10.1016/j.it.2020.04.004

[9] a) G. Zhou , R. Myers , Y. Li , Y. Chen , X. Shen , J. Fenyk‐Melody , M. Wu , J. Ventre , T. Doebber , N. Fujii , N. Musi , M. F. Hirshman , L. J. Goodyear , D. E. Moller , J. Clin. Invest. 2001, 108, 1167; 11602624 10.1172/JCI13505 PMC209533

[10] a) L. Ding , G. Liang , Z. Yao , J. Zhang , R. Liu , H. Chen , Y. Zhou , H. Wu , B. Yang , Q. He , OncoTargets Ther. 2015, 6, 36441; 10.18632/oncotarget.5541 PMC4742188 26497364

[11] J.‐H. Cha , W.‐H. Yang , W. Xia , Y. Wei , L.‐C. Chan , S.‐O. Lim , C.‐W. Li , T. Kim , S.‐S. Chang , H.‐H. Lee , J. L. Hsu , H.‐L. Wang , C.‐W. Kuo , W.‐C. Chang , S. Hadad , C. A. Purdie , A. M. McCoy , S. Cai , Y. Tu , J. K. Litton , E. A. Mittendorf , S. L. Moulder , W. F. Symmans , A. M. Thompson , H. Piwnica‐Worms , C.‐H. Chen , K.‐H. Khoo , M.‐C. Hung , Mol. Cell 2018, 71, 606. 30118680 10.1016/j.molcel.2018.07.030 PMC6786495

[12] a) S. Verdura , E. Cuyàs , B. Martin‐Castillo , J. A. Menendez , Oncoimmunology 2019, 8, 1633235; 10.1080/2162402X.2019.1633235 PMC6791450 31646077

[13] H. Tian , G. Wang , W. Sang , L. Xie , Z. Zhang , W. Li , J. Yan , Y. Tian , J. Li , B. Li , Y. Dai , Nano Today 2022, 43, 101405.

[14] a) Q. Chen , C. Liang , X. Sun , J. Chen , Z. Yang , H. Zhao , L. Feng , Z. Liu , Proc. Natl. Acad. Sci. U. S. A. 2017, 114, 5343; 28484000 10.1073/pnas.1701976114 PMC5448233

