# Supplementary material for: Self‐Degradable Nanogels Reshape Immunosuppressive Tumor Microenvironment via Drug Repurposing Strategy to Reactivate Cytotoxic CD8+ T Cells
Source: Adv Sci (Weinh). 2023 May 5;10(21):2301661. doi: 10.1002/advs.202301661 (PMC10375179; doi:10.1002/advs.202301661)
Supplement: Supplementary file 1 — Supporting Information [file ADVS-10-2301661-s001.pdf]

ADVANCED SCIENCE
Open Access

Supporting Information

for *Adv. Sci.*, DOI 10.1002/advs.202301661

Self-Degradable Nanogels Reshape Immunosuppressive Tumor Microenvironment via Drug Repurposing Strategy to Reactivate Cytotoxic $CD8^+$ T Cells

*Hao Tian, Wenxi Li, Guohao Wang, Ye Tian, Jie Yan, Songtao Zhou, Xinying Yu, Bei Li and Yunlu Dai**

Supporting Information

Self-Degradable nanogels reshape immunosuppressive tumor microenvironment via drug repurposing strategy to reactivate cytotoxic CD8$^+$ T cells

*Hao Tian, Wenxi Li, Guohao Wang, Ye Tian, Jie Yan, Songtao Zhou, Xinying Yu, Bei Li and Yunlu Dai**

1. Materials and Methods

1.1. Materials

Poly(ethylene glycol) Methyl Ether, average M.W. 5000 was purchased from J&K Scientific. IR-780 iodide was purchased from Sigma-Aldrich. All other chemical agents were purchased from Aladdin. Annexin V-FITC/PI Apoptosis Detection Kit was purchased from BD biosciences. IL-4, IFN-γ, recombinant mouse macrophage colony-stimulating factor (M-CSF) and recombinant mouse granulocyte-macrophage colony-stimulating factor (GM-CSF) were purchased from Peprotech. AMPKα and AMPKα Thr172-p were purchased from Cell Signaling Technology. DNase I was purchased from Roche. Collagenase (Type IV) powder was purchase from Gibco. Phosphate buffered saline (PBS) were purchased from Thermo Fisher Scientific. Anti-CD8 antibody (clone YTS 169.4) was purchased Bio X Cell and anti-PD-1 antibody from Leinco.

Antibodies used for flow cytometry analysis were as follows. Anti-mouse PD-L1 (clone number: 10F.9G2, Catalog: 124301), APC-Granzyme B recombinant antibody (clone: QA16A02, Catalog: 372204), FITC-Ki67 antibody (clone: 16A8, Catalog: 652410) and PerCP/Cyanine5.5 Tim-3 antibody (clone: RMT3-33, Catalog:119718) was purchased by Biolegend. FITC-CD45 monoclonal antibody (clone: 30-F11, Catalog: 11-0451-81), PerCP-Cyanine5.5- CD45 monoclonal antibody (clone: 30-F11, Catalog: 11-0451-81),

FITC-CD3e monoclonal antibody (clone: 145-2C11, Catalog:11-0031-82),

PE-CD3e monoclonal antibody (clone 145-2C11, Catalog: 12-0031-82), PE-CD4 monoclonal antibody (clone GK1.5, Catalog: 11-0041-82), PE-CD8 monoclonal antibody (clone: 53-6.7, Catalog: 12-0081-82), PerCP-Cyanine5.5-CD8a monoclonal antibody (clone 53-6.7, Catalog: 45-0081-82), PE-FOXP3 Monoclonal Antibody (clone FJK-16s, Catalog: 12-5773-82), FITC-CD11b monoclonal antibody (clone M1/70, Catalog: 11-0112-81), PerCP-Cyanine5.5- F4/80 monoclonal antibody (clone: BM8, Catalog: 15-4801-82), FITC-CD11c monoclonal antibody (clone N418, Catalog: 11-0114-82), PE-CD86 monoclonal antibody (clone GL1, Catalog: 12-0862-81), APC-CD80 monoclonal antibody (clone 16-10A1, Catalog:17-0801-81), and flow cytometry staining buffer (FCSB) were purchased from eBioscience. Foxp3/transcription factor fixation/permeabilization concentrate and diluent, permeabilization buffer were purchased from Invitrogen.

1.2. Cell lines and animals.

Murine 4T1 breast cancer cells and RAW264.7were purchased from the American Type Culture Collection. BALB/c mice (6-8 weeks, 18 ± 2g) were purchased from the Animal Research Core of University of Macau. All animal procedures were performed following an approved protocol (UMARE-030-2018) by the University of Macau Animal Ethics Committee.

1.3. Characterization.

NMR spectra was conducted on Nuclear Magnetic Resonance (NMR) spectrometer (BRUKER, AscendTM 400 MHz). Dynamic diameters were determined by Malvern Zetasizer Nano ZSP system ZEN5600. The absorption spectra were detected by a Shimadzu UV-1800 UV–Vis–NIR spectrophotometer. The transmission electron microscopy (TEM) images and energy dispersive X-ray spectroscopy (EDS) mapping images were acquired on a JEM-3010UHR/JEM-2100F field emission electron microscope. Confocal microscopy images were acquired by Carl Zeiss LSM710 confocal microscope. Flow cytometry analyses were performed by CytoFLEX flow cytometer (Beckman).

1.4. Polymer synthesis

1.4.1. Synthesis of the 3,3'-(propane-2,2-diylbis(sulfanediyl))dipropionic acid (ROS linker)

The mercaptoacetic acid (5.3 g, 50 mmol), acetone (1.45 g, 25 mmol) and 10 μL trifluoroacetic acid were dissolved in 10 mL dichloromethane (DCM, CH_2Cl_2). Stir the mixture at room temperature for 12 h. Then the product was purified by column chromatography (eluent: CH_2Cl_2/ CH_3OH, 20/1, v/v). Yield: 4.32 g (64 %)

1.4.2. Synthesis of the PEG-ROS

The Synthesis of PEG-b-NH_2 was according to our previous method. Meanwhile, the ROS linker (252 mg, 1 mmol) was dissolved in 2ml of thionyl chloride ($SOCl_2$) and stir at room temperature for 12 hours. Then evaporated $SOCl_2$ by rotary evaporate to get solid product whose carboxyl groups has changed to acid chloride. Finally, the PEG-b-NH_2 (700 mg) and acid chloride functionalized ROS linker (28.9 mg, 0.1mmol) in dichloromethane (5 mL) and stirred for 12 h. Then, most of the solvents were evaporated using a vacuum rotary. The remaining solution was precipitated into an excess of diethyl ether to generate pale residues, the residues were dried in a vacuum oven overnight at room temperature. Yield: 640 mg.

1.4.3. Synthesis of the Met-Pho

Metformin hydrochloride (1.29 g, 10 mmol) and P-phthalaldehyde (1.34 g, 10 mmol) were dissolved in 15 mL MeOH, then reacted overnight at 60 °C and under the nitrogen atmosphere. Finally, the product was purified by column chromatography (eluent: CH_2Cl_2/ CH_3OH, 20/1, v/v). Yield: 1.51 g (57 %)

1.4.4. Synthesis of the PM

A solution of PEG-ROS (300 mg), Met-Pho (245 mg) in 20 mL MeOH was stirred under nitrogen at 60 ℃ for overnight. Then the solvent was removed by a rotary evaporator and further precipitated the crude product in diethyl ether three times. Then the products and 3,4-dihydroxybenzaldehyde (270 mg, 2.0 mmol) were reacted under the same conditions to obtain the final product PM (385 mg).

1.4.5. Synthesis of the PM-IR

PM (400 mg), IR 780 (30 mg, 45 μmol) were dissolved in DMF, and triethylamine (200 μL) was added to this solution. The reaction was stirred overnight at 40 ℃. After dialysis (MWCO, 3.5 kDa) for 2 days against distilled water in a dark environment, the solution was lyophilized to afford solid products. Yield: 210 mg (49 %). Confirming the reaction is complete by the changing maximum absorption wavelength from the product.

1.5. Cytotoxicity studies in vitro

The cell viability of 4T1 cells were assessed by MTT assay, which seeded in 96-well plates at a density of 5000 cells per well for 24 h. Then a series solution of Imi, PM, and PMI with different concentrations were added to the medium, and the cells were incubated for another 24 h. Then the wells were incubated with MTT medium for 4 h and monitored the absorbance at 490 nm by a microplate reader (PerkinElmer Vivtor X3 Multimode Plate Reader). Cell viability (%) = (OD490 sample - OD490 blank) / (OD490 control - OD490 blank) ×100 %.

Apoptosis flow cytometric measurement was conducted by seeded 4T1 cells (5×10^4 per well) in 12-well plates and incubated with Imi, PM, and PMI at the Imi dosage of 10 μg mL^{-1} and PM dosage of 250 μg mL^{-1} for 24 h. Annexin V-FITC/PI Apoptosis Detection Kit was used for cell staining according to the protocol provided by the manufacturer.

1.6. Flow Cytometry Analysis

Tumor immune microenvironment analysis was using the same 4T1 orthotopic tumor model, the procedures of tumor inoculation and subsequent treatments remained the anti-tumor effect evaluation experiment. After sacrificing the tumor-bearing mice, the tumor-draining lymph nodes (TDLNs) and tumor tissues were dissected for immunological evaluation following the standard protocol. TDLNs were cut into small pieces and mechanically minced against a 70 μm cell strainer to obtain single cell suspension and washed with FACS buffer. Tumors were incubated in dissociation buffer with 1640 medium containing hyaluronidase (100 U), deoxyribonuclease (100 mg mL^{-1}), and collagenase IV (1 mg mL^{-1}) at 37 °C for 1.5 h

for digesting tissue. And the tissue was also mechanically minced against a 70 μm cell strainer and washed with FACS buffer. Then, all cells were fixed using the Foxp3 Transcription Factor Fixation/Permeabilization Concentrate and Diluent solutions (Invitrogen) and Permeabilization Buffer (Invitrogen) following the manufacturer's specifications. To investigate the DC maturation, the cells were further stained with corresponding antibodies: CD11c, CD80, CD86 following the manufacturer's instructions. For M1/M2-like macrophages analysis, cells were stained with CD11b, F4/80, CD206 and CD80. To analyze T cells were stained with corresponding antibodies: CD45, CD4, CD8, IFN-γ, Granzyme B, Ki67, Tim-3, following the manufacturer's instructions. The stained cells were detected using flow cytometry. The data were analyzed using FlowJo 10.0.

1.7. Enzyme-Linked Immunosorbent Assay.

Cytokines Expression level (TNF-α, IL-6, IL-10 and IL-12p70) from culture medium and serum were detected by commercial ELISA kits (Neobioscience Biotechnology, Shenzhen, China) following the protocol.

2. Figures and figure captions

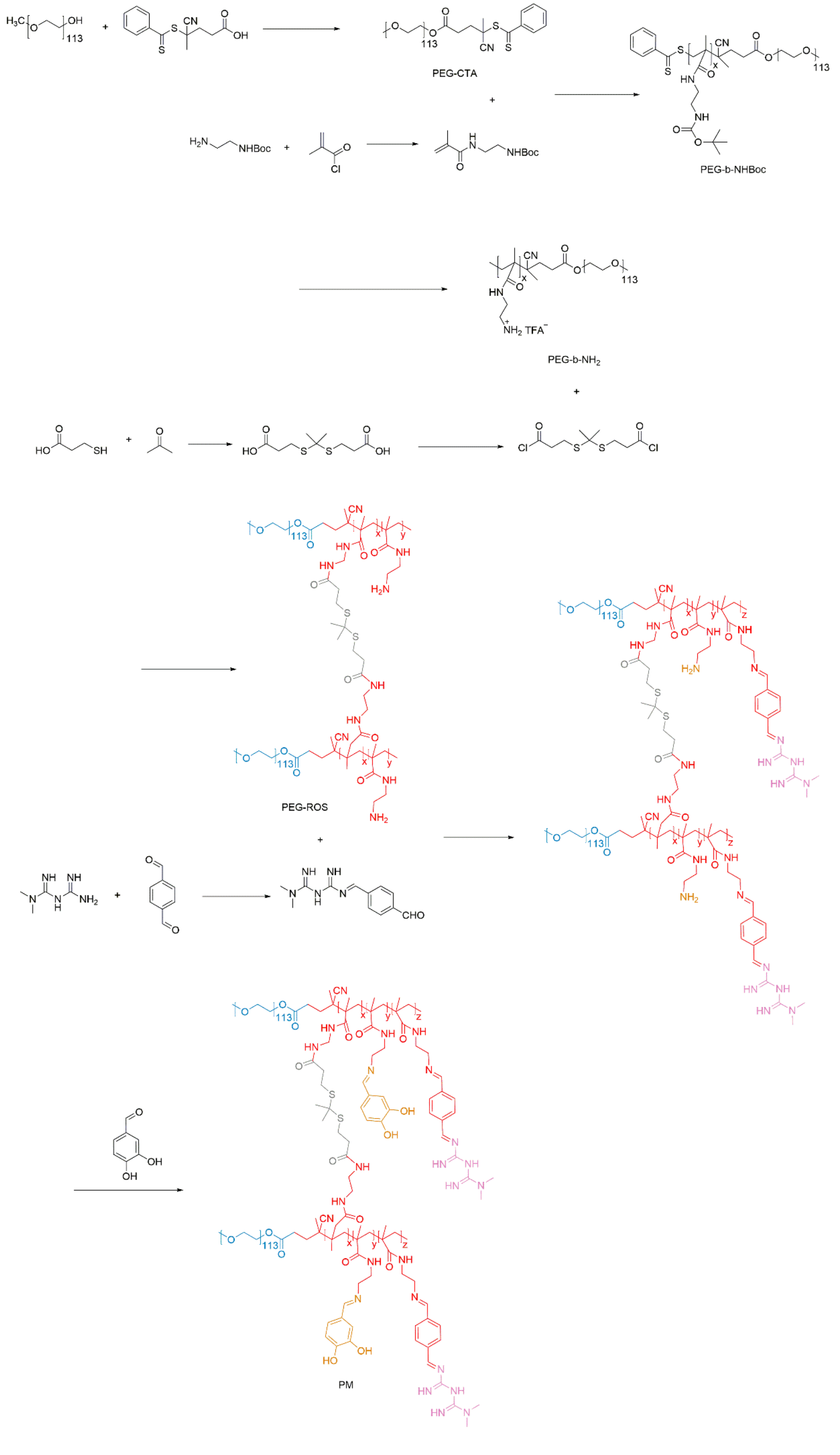
PEG-CTA
PEG-b-NHBoc
PEG-b-NH2
PEG-ROS
PM

Figure S1. The synthesis route of ROS-responsive phenolic polymer.

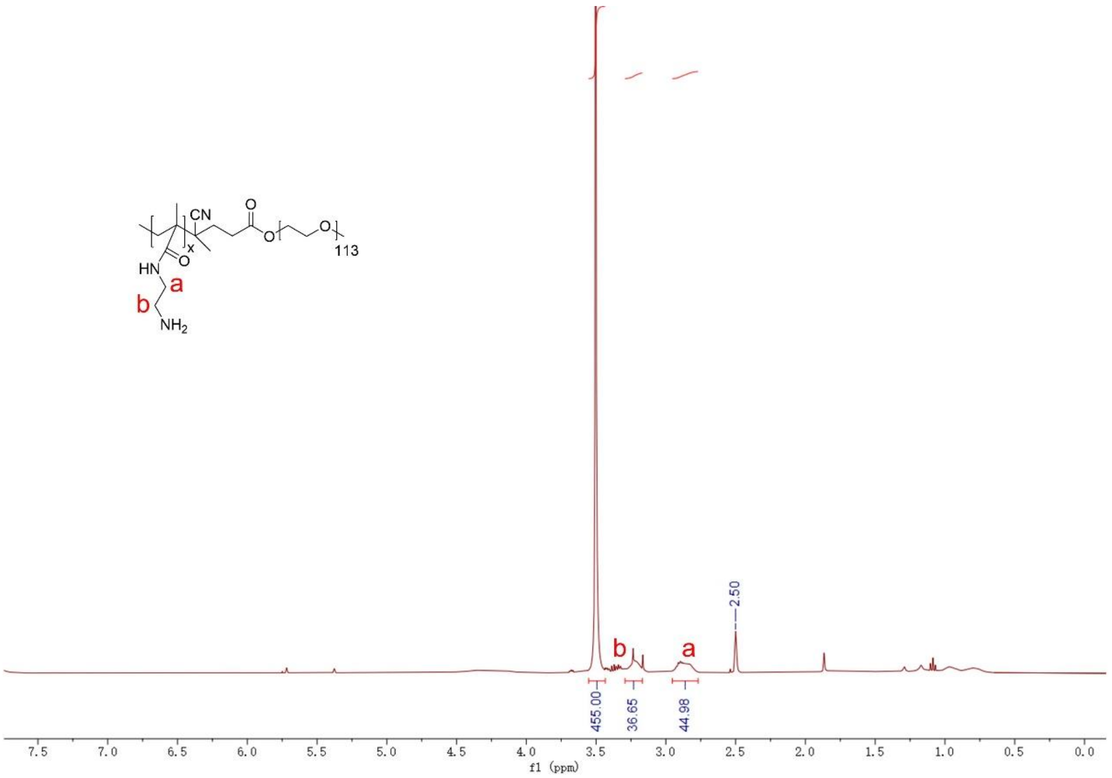

Figure S2. ^{1}H NMR spectrum of the PEG-NH_2 in DMSO-d6.

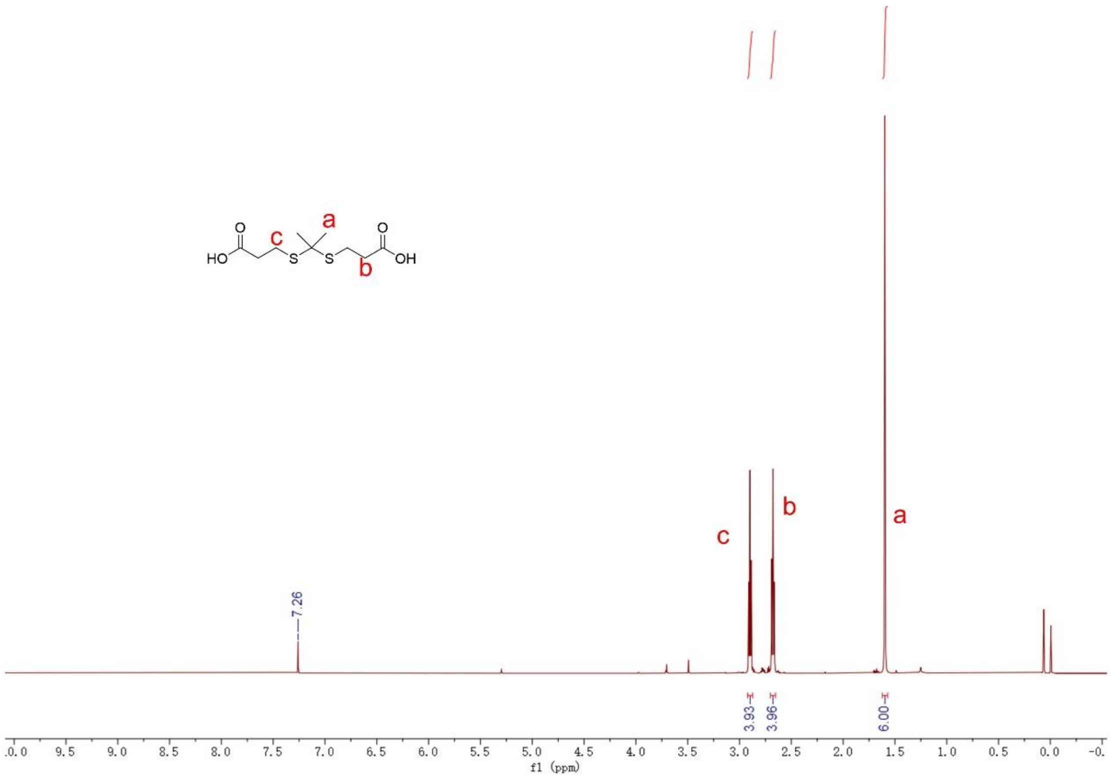

Figure S3. [1] H NMR spectrum of the 3,3'-(propane-2,2-diylbis(sulfanediyl))dipropionic acid in DMSO-d6.

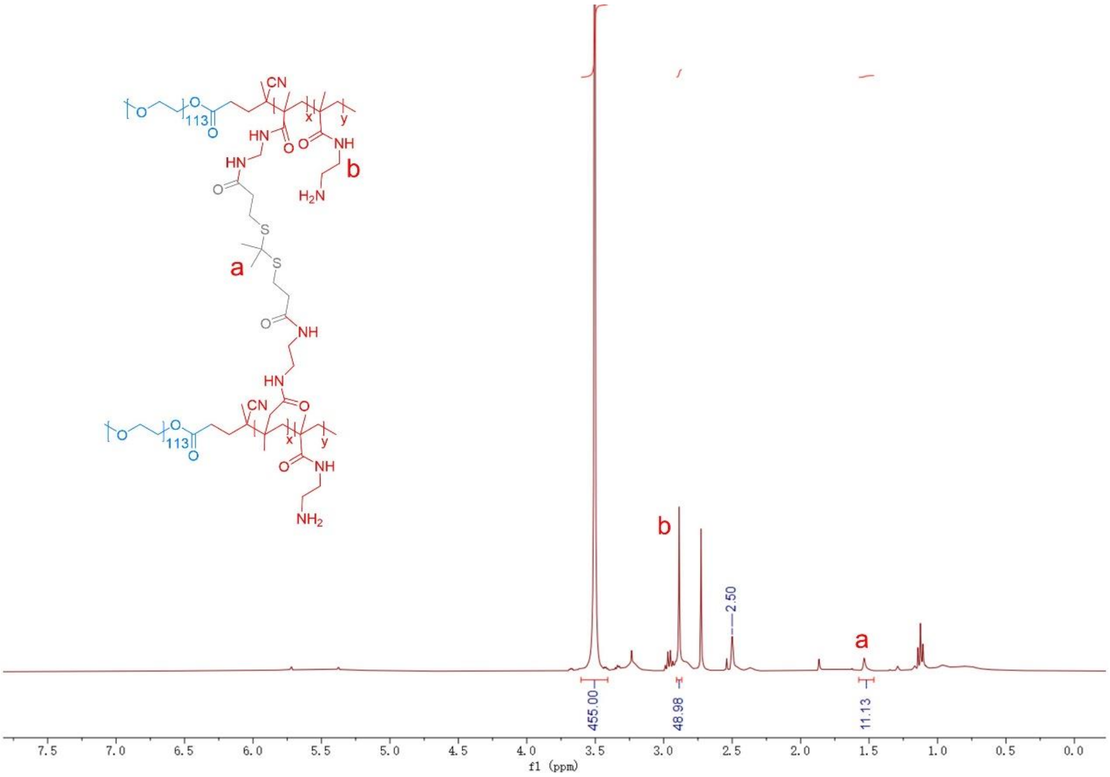

Figure S4. [1] H NMR spectrum of the PEG-ROS in DMSO-d6.

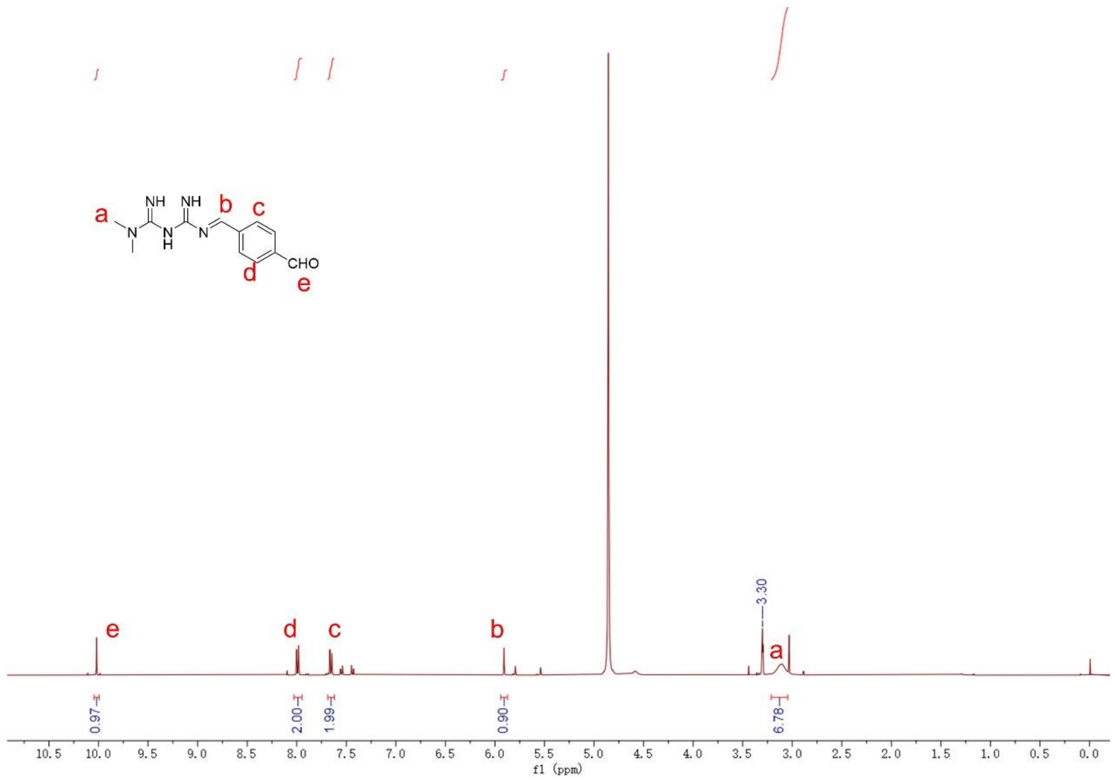

Figure S5. ^{1}H NMR spectrum of the Met-Pho in CD_3OD.

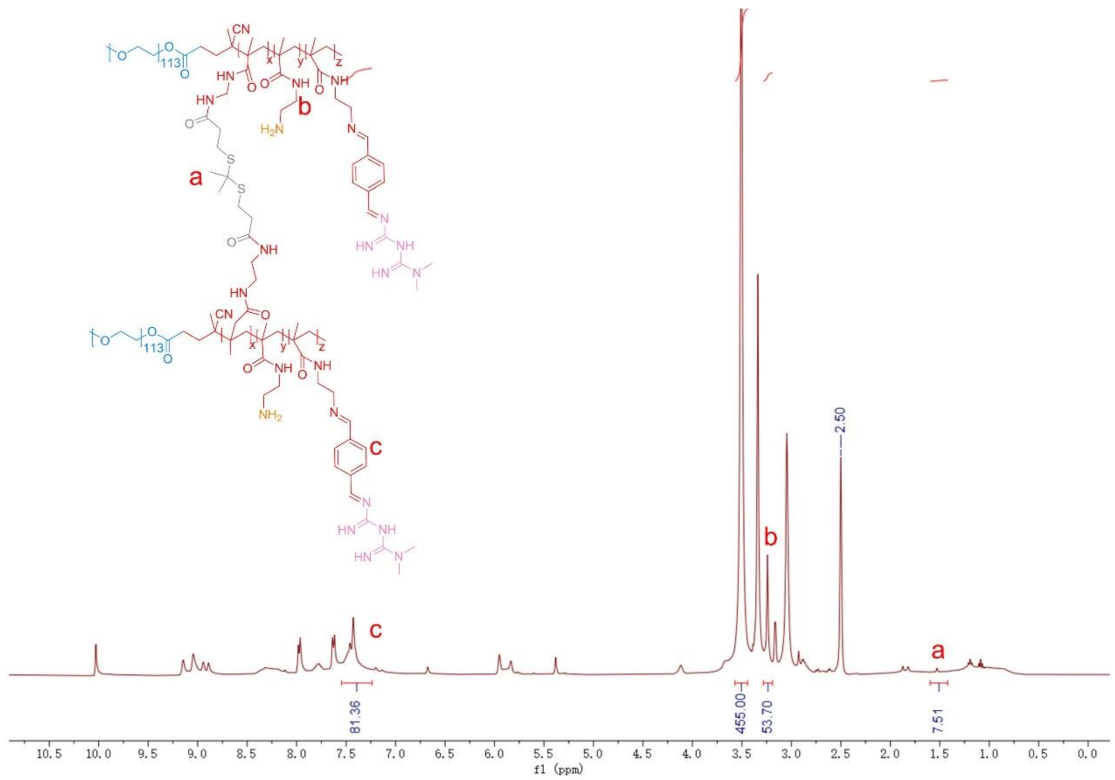

Figure S6. ^{1}H NMR spectrum of the PEG-ROS-Met in DMSO-d6.

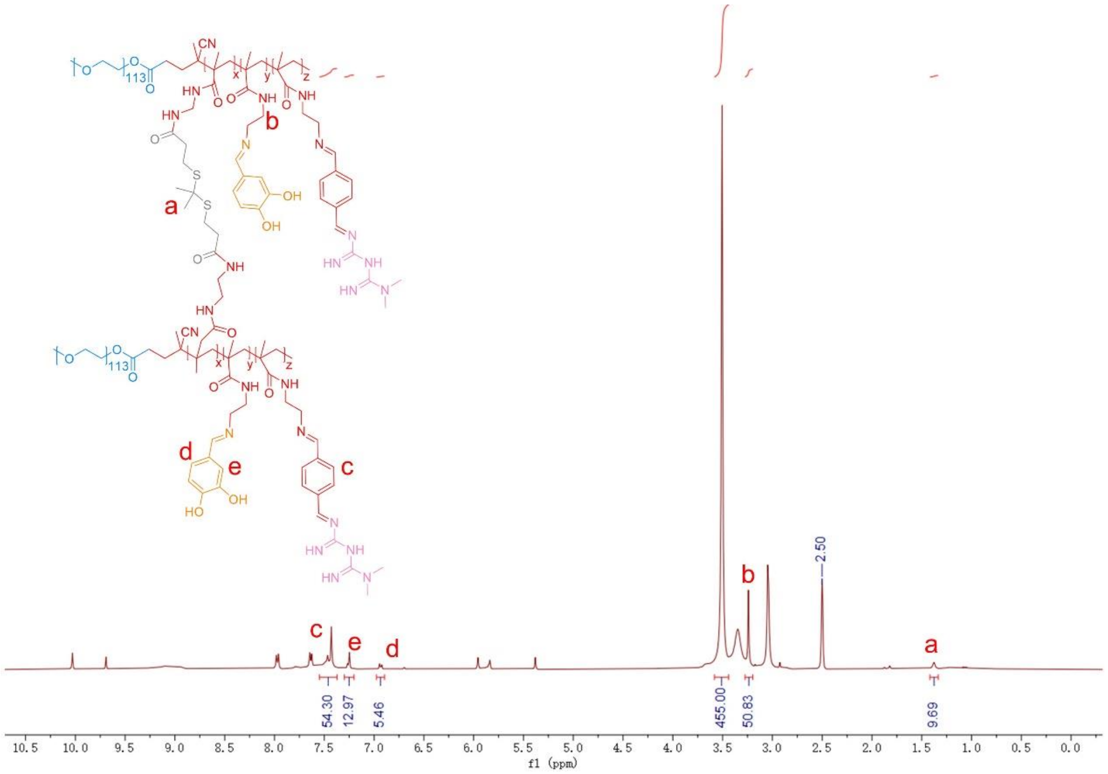

Figure S7. [1] H NMR spectrum of the PM in DMSO-d6.

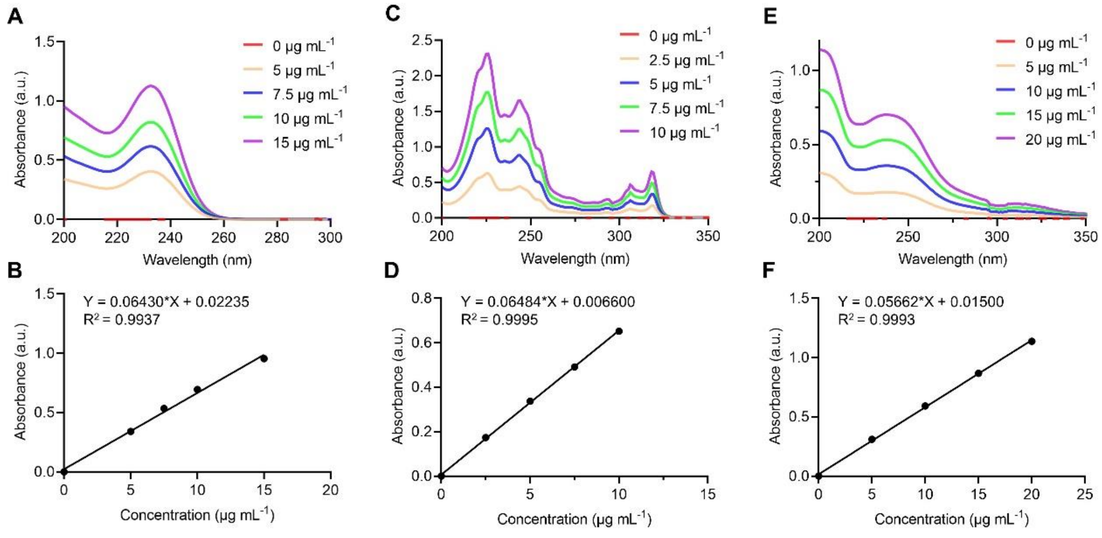

Figure S8. A) The absorbance spectra of Met at a series of concentrations. B) Standard curve of Met at 245 nm. C) The absorbance spectra of Imi at a series of concentrations. D) Standard curve of Imi at 318 nm. E) The absorbance spectra of PM at a series of concentrations. F) Standard curve of PM at 200 nm.

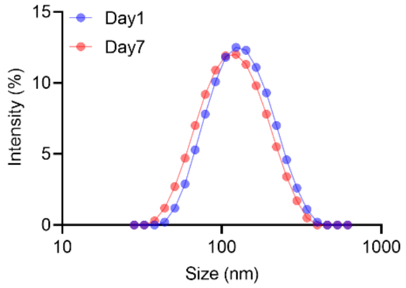

Figure S9. Dynamic light scattering (DLS) result of PMI nanogels at day 1 and day 7.

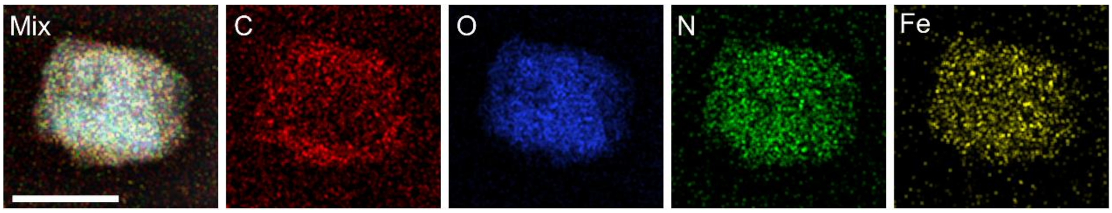

Figure S10. Energy-dispersive X-ray spectroscopy (EDS) analysis of PMI nanogels. Scale bar: 200 nm.

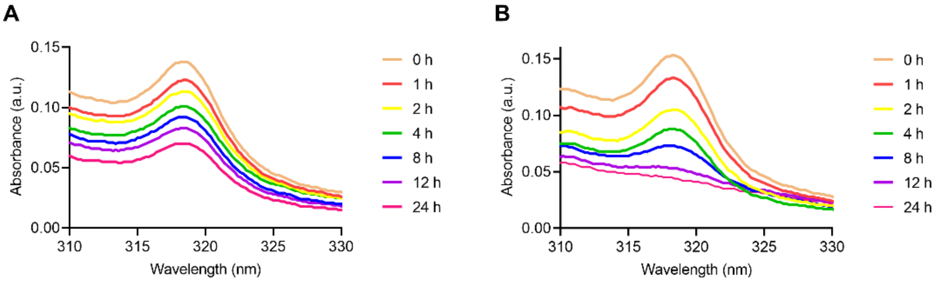

Figure S11. A, B) Absorbance spectra at different time points after incubation in pH 7.4 (A) and pH 6.8 solution containing 100 μM H_2O_2 (B) solutions to evaluate Imi release profiles.

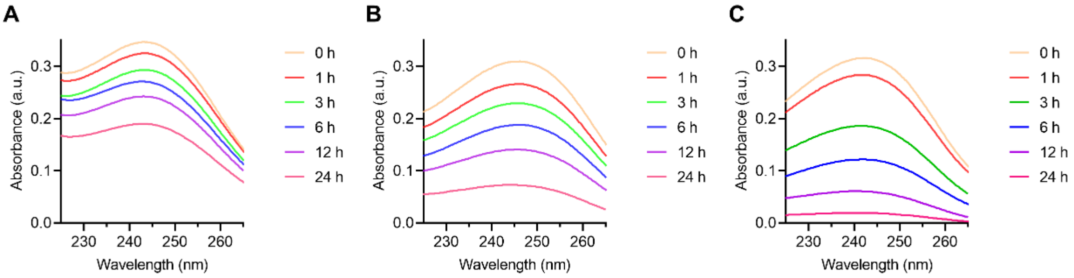

Figure S12. A-C) Absorbance spectra at different time points after incubation in pH 7.4 (A), 6.8 (B), and 5.5 (C) solutions to evaluate Met release profiles.

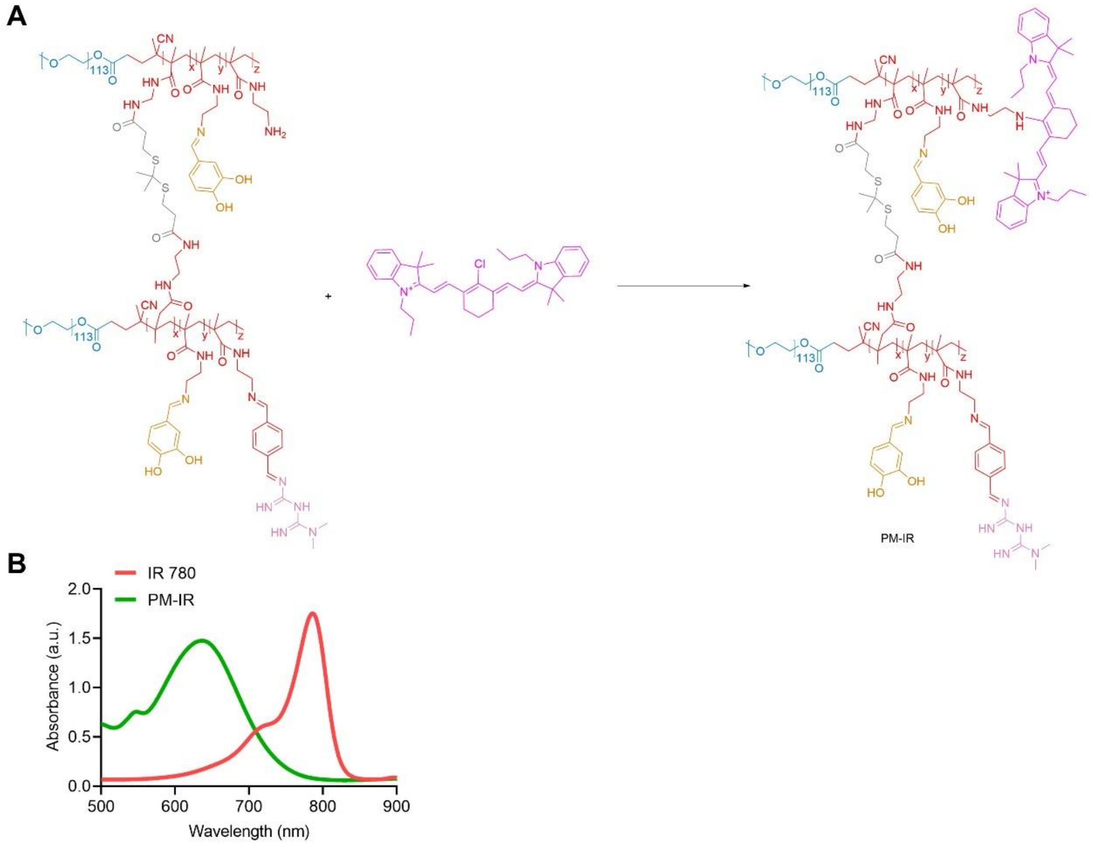

Figure S13. Synthesis of PM-IR. A) Chemical structure and synthesis route of PM-IR. B) The UV-Vis absorption spectra of IR 780 and PM-IR in DMSO/Water (50:50, v:v). IR 780 is a fluorescent dye with maximum absorption at 780nm. Its conjugation to the polymer PM will cause a blue shift in their absorption. Therefore, we confirmed the completion of the reaction by determining the maximum absorption wavelength of the product.

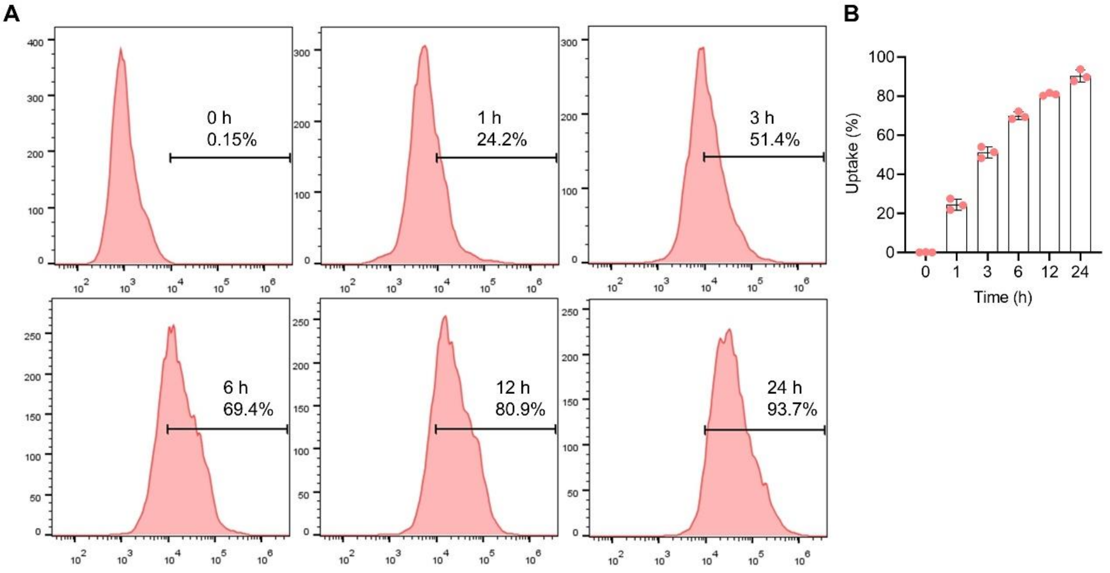

Figure S14. A, B) Flow cytometric and quantitative analyses of 4T1 cells incubated with PMI-IR nanogels at different time points. Data are presented as mean values ± SD (n = 3).

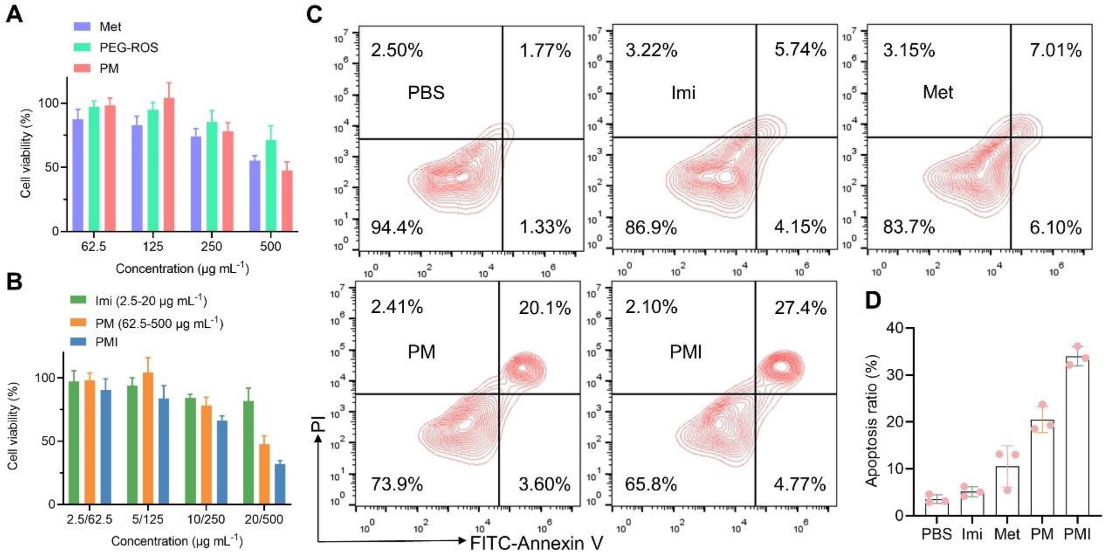

Figure S15. A, B) Cytotoxicity of free Met, PEG-ROS, PM, Imi, and PMI in 4T1 cells after incubating for 24 h. C, D) Representative flow cytometry images and quantitative analyses of cell apoptosis undergoing different treatments. Data are presented as mean values ± SD (n = 3).

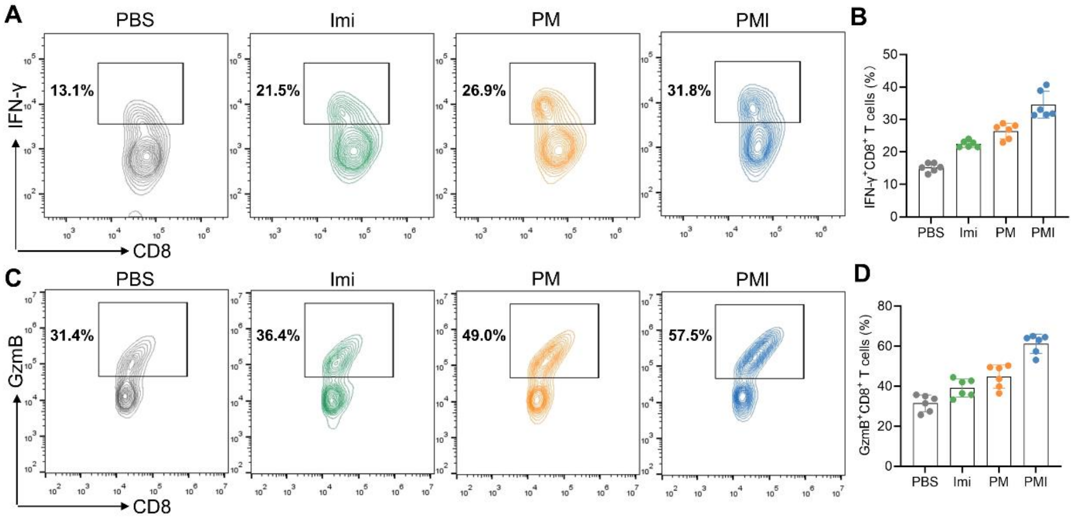

Figure S16. A-D) Flow cytometric analysis of IFN-γ^+ (A, B) and GzmB$^+$ (C, D) in CD8$^+$ T cells. Data are presented as mean values ± SD (n = 6).

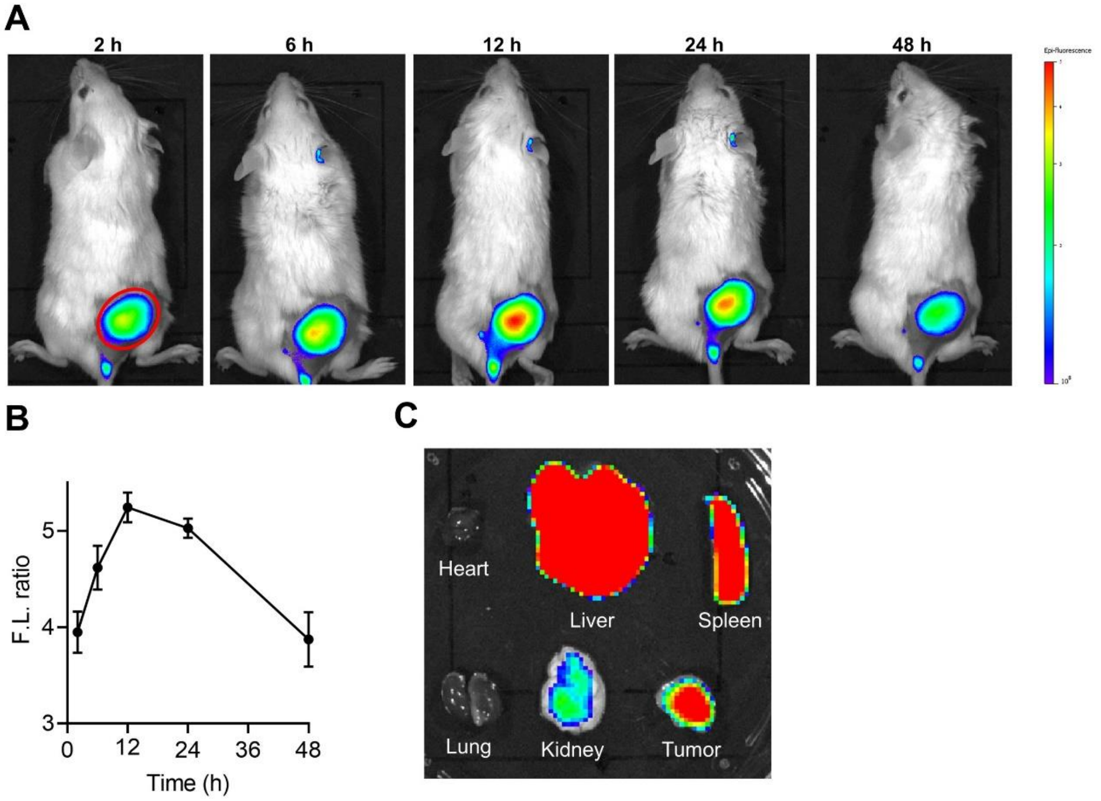

Figure S17. A) In vivo fluorescent images of 4T1 tumor-bearing mice at 2, 6, 12, 24, and 48 h after i.v. injection of PMI nanogels. The red circle indicated the location of the tumor. B) Quantification of the corresponding fluorescence intensity at different time points. Data are presented as mean values ± SD (n = 3). C) The ex vivo fluorescent images of main organs and tumors at 12 h.

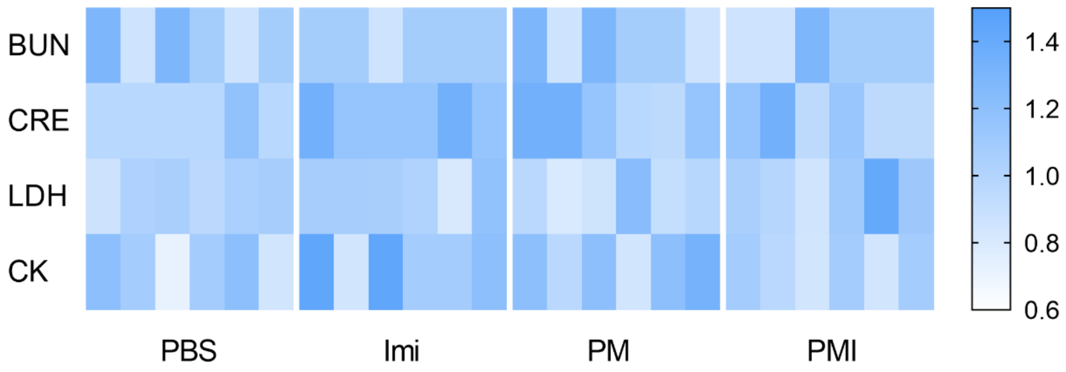

Figure S18. Biochemical parameters include blood urea nitrogen (BUN), creatinine (CRE), lactate dehydrogenase (LDH), and creatine kinase (CK) were determined. The color variance represents a ratio of the mice's biochemical data versus, n=6.

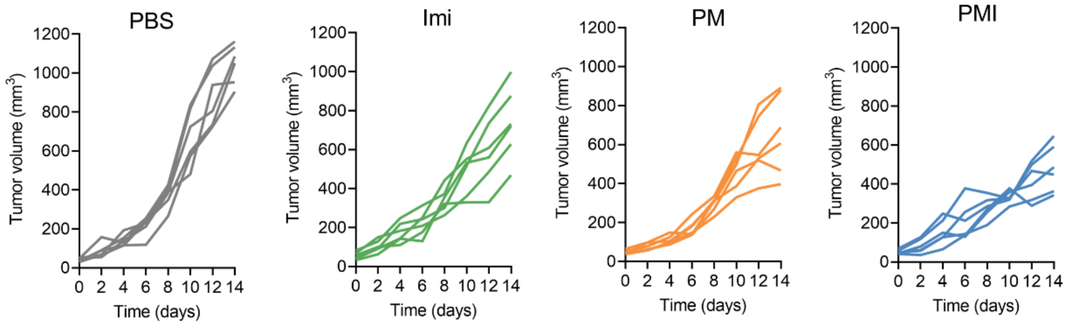

Figure S19. Individual tumor growth curves.

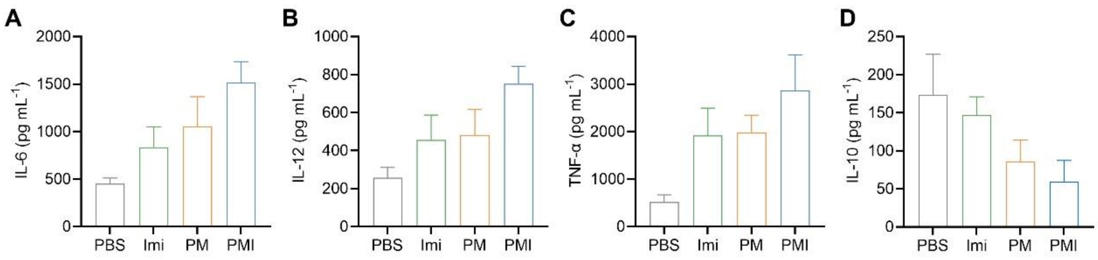

Figure S20. ELISA analysis of proinflammatory cytokine levels of IL-6 (A), IL-12 (B), TNF-α (C) and IL-10 (D) in serum.

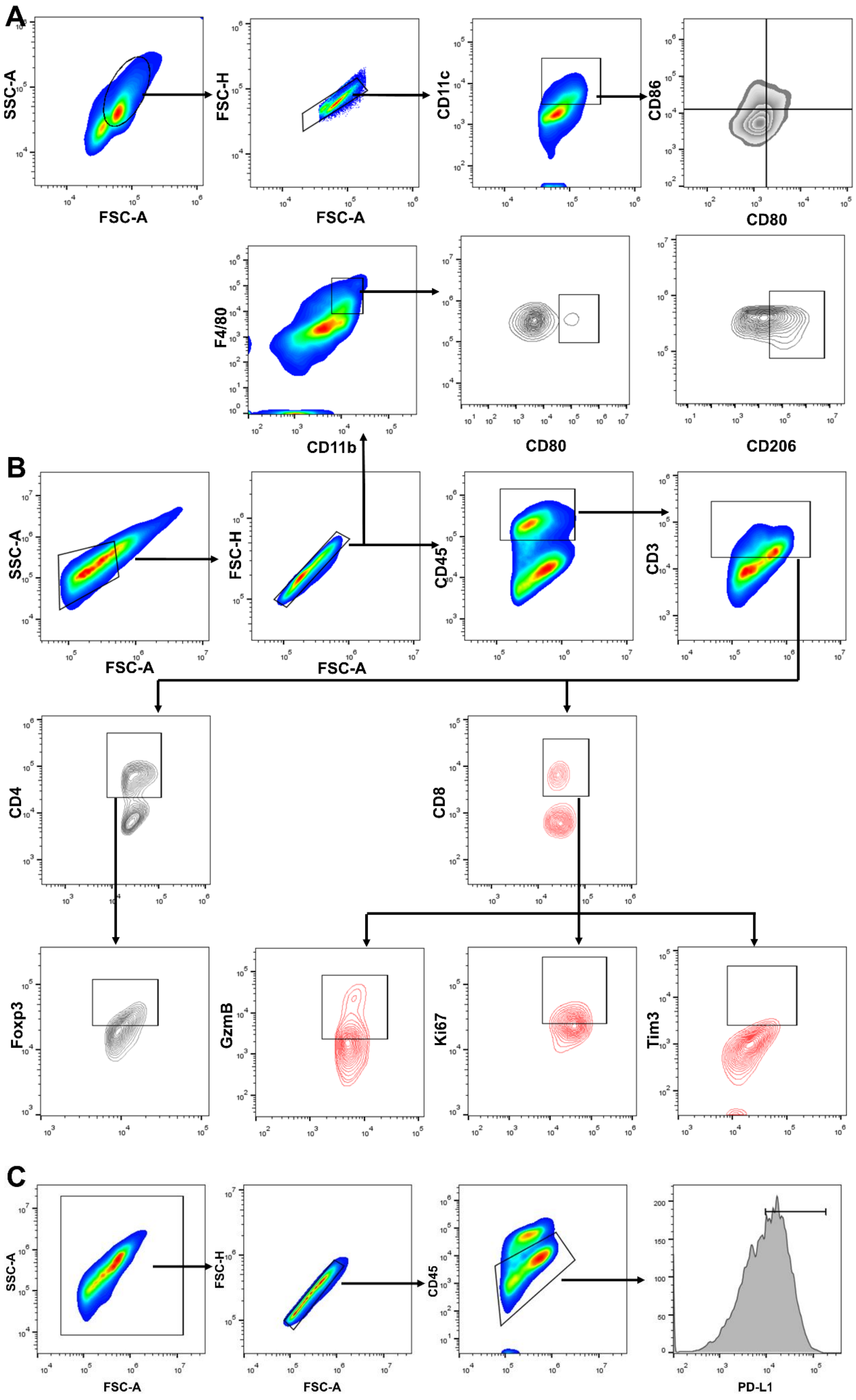

A
SSC-A
FSC-A
FSC-H
FSC-A
CD11c
CD86
CD80
F4/80
CD11b
CD80
CD206
B
SSC-A
FSC-A
FSC-H
FSC-A
CD45
CD3
CD4
CD8
Foxp3
GzmB
Ki67
Tim3
C
SSC-A
FSC-A
FSC-H
FSC-A
CD45
PD-L1

Figure S21. Gating strategy for identifying major immune cells. A) Gating strategies for mature DCs (CD11c$^+$CD80$^+$CD86$^+$) in tumor-draining lymph nodes. B) Gating strategies for identifying immune cells in tumors, including M1- and M2-like TAMs (CD11b$^+$F4/80$^+$CD80$^+$ or CD206$^+$), CD4$^+$ T cells (CD45$^+$CD3$^+$CD4$^+$), Treg cells (CD45$^+$CD3$^+$CD4$^+$Foxp3$^+$), CD8$^+$ T cells (CD45$^+$CD3$^+$CD8$^+$), GzmB$^+$CD8$^+$ T cells (CD45$^+$CD3$^+$CD8$^+$GzmB$^+$), Ki67$^+$CD8$^+$ T cells (CD45$^+$CD3$^+$CD8$^+$Ki67$^+$), and Tim3$^+$CD8$^+$ T cells (CD45$^+$CD3$^+$CD8$^+$Tim3$^+$). C) Gating strategy for PD-L1 in cancer cells.

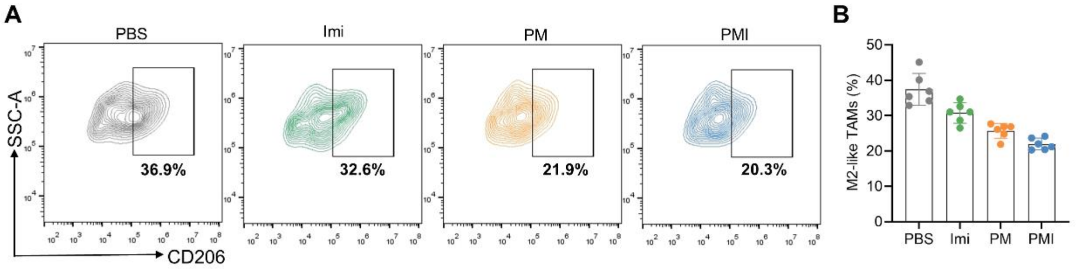

Figure S22. Flow cytometric and quantitative analyses of M2-like TAMs after various treatments. Data are presented as mean values ± SD (n = 6).

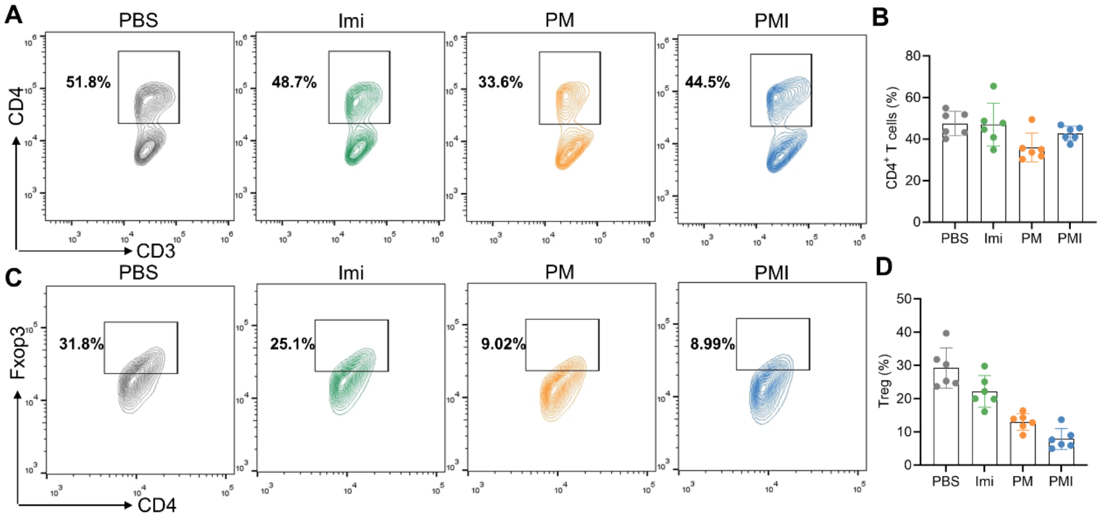

Figure S23. A, B) Representative flow cytometric images and quantitative analyses of CD4$^+$ T cells after different treatments. C, D) Representative flow cytometric images and quantitative analyses of Treg cells after different treatments. Data are presented as mean values ± SD (n = 6).

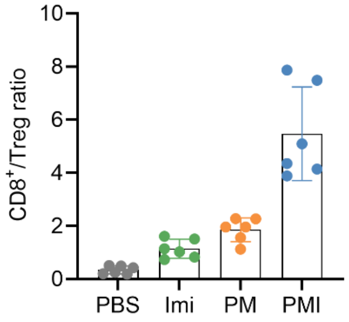

Figure S24. The intratumoral ratio of CD8$^+$ T cells to Treg after different trentments. Data are presented as mean values ± SD (n = 6).

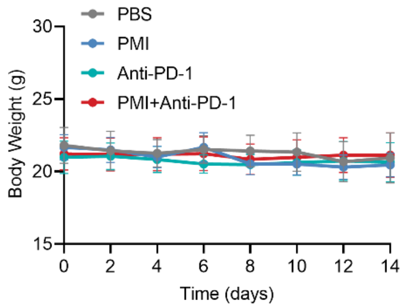

Figure S25. The body weight of mice during the treatment.

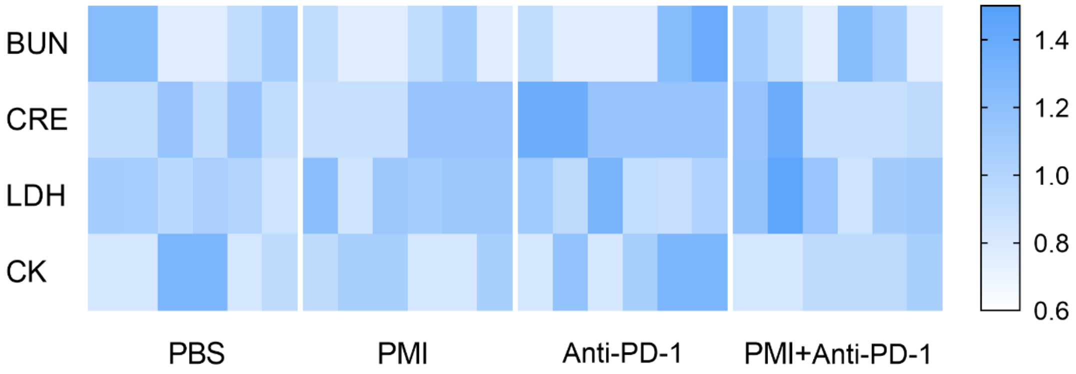

Figure S26. Biochemical parameters include blood urea nitrogen (BUN), creatinine (CRE), lactate dehydrogenase (LDH), and creatine kinase (CK) were determined. The color variance represents a ratio of the mice's biochemical data versus, n=6.

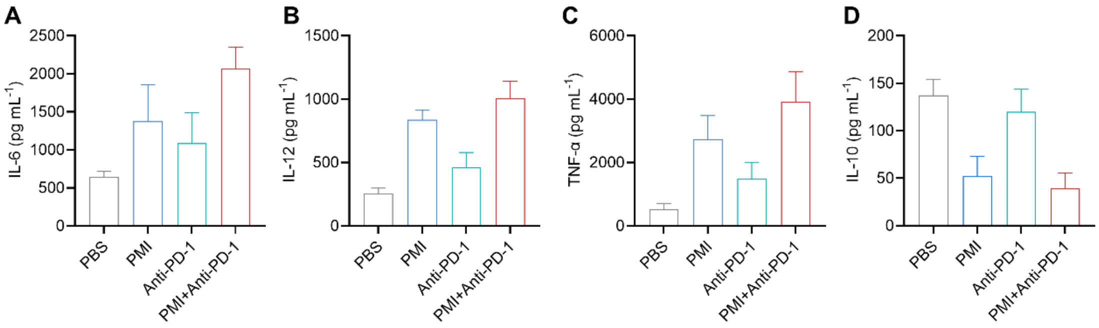

Figure S27. ELISA analysis of proinflammatory cytokine levels of IL-6 (A), IL-12 (B), TNF-α (C) and IL-10 (D) in serum.

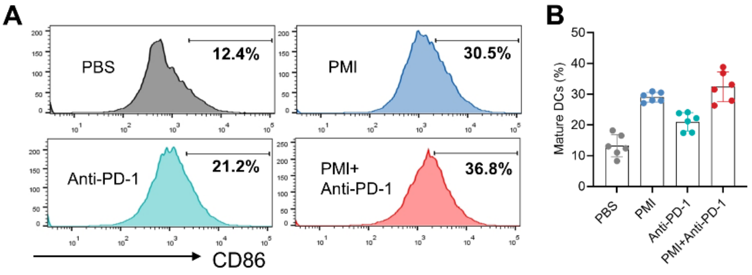

Figure S28. A, B) Flow cytometric and quantitative analyses of mature DCs in TDLNs of mice after different treatments. Data are presented as mean values ± SD (n = 6).

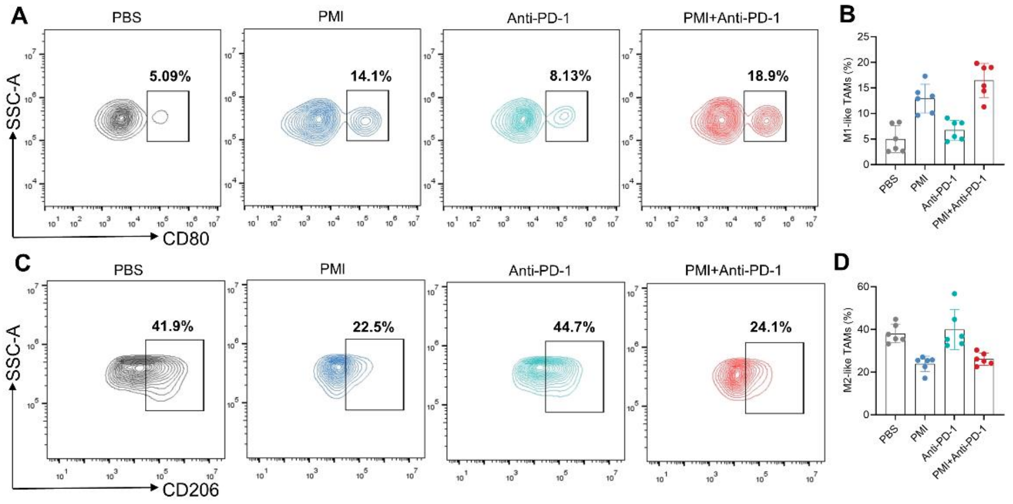

Figure S29. A, B) Flow cytometric and quantitative analyses of M1-like TAMs after different treatments. C, D) Flow cytometric and quantitative analyses of M2-like TAMs after different treatments. Data are presented as mean values ± SD (n = 6).

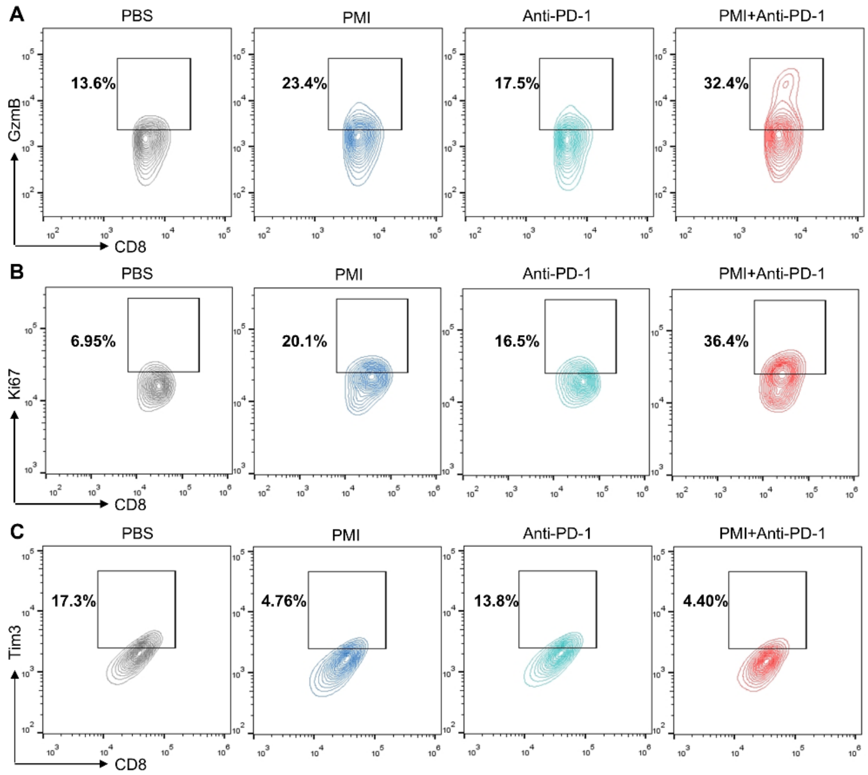

Figure S30. A) Representative flow cytometry plots of GzmB$^+$CD8$^+$ T cells. B) Representative flow cytometry plots of Ki67$^+$CD8$^+$ T cells. C) Representative flow cytometry plots of Tim3$^+$CD8$^+$ T cells.

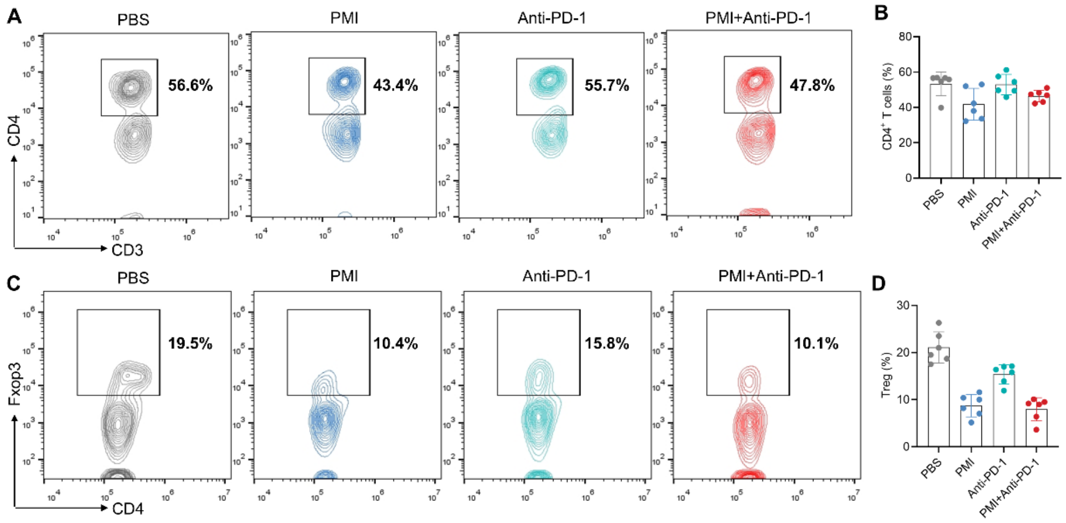

Figure S31. A, B) Representative flow cytometric images and quantitative analyses of CD4$^+$ T cells. C, D) Representative flow cytometric images and quantitative analyses of Treg cells.
